# Macrophage correlates with immunophenotype and predicts anti-PD-L1 response of urothelial cancer

**DOI:** 10.7150/thno.46176

**Published:** 2020-05-25

**Authors:** Dongqiang Zeng, Zilan Ye, Jiani Wu, Rui Zhou, Xinxiang Fan, Gaofeng Wang, Yiqiang Huang, Jianhua Wu, Huiying Sun, Miaohong Wang, Jianping Bin, Yulin Liao, Nailin Li, Min Shi, Wangjun Liao

**Affiliations:** 1 Department of Oncology, Nanfang Hospital, Southern Medical University, Guangzhou, Guangdong, P. R. China;; 2 Department of Urology, Sun Yat-sen Memorial Hospital, Sun Yat-sen University, Guangzhou, China;; 3 Department of Dermatology, Johns Hopkins School of Medicine, Baltimore, Maryland, USA;; 4 Department of Urology, Daping Hospital, Army Medical University, Chongqing, P.R. China;; 5 Department of Cardiology, State Key Laboratory of Organ Failure Research, Nanfang Hospital, Southern Medical University, Guangzhou, China;; 6 Karolinska Institutet Department of Medicine-Solna, Clinical Pharmacology Group, Karolinska University Hospital-Solna, 171 76, Stockholm, Sweden.

**Keywords:** immune checkpoint blockade, macrophage, immunophenotype, urothelial cancer

## Abstract

Immune-checkpoint blockades (ICBs) have been routinely implemented to treat metastatic urothelial cancer (mUC), whereas robust biomarkers are urgently warranted. Herein, we explored latent promising biomarkers based on 348 pretreatment mUC samples from IMvigor210.

**Methods:** The genome, transcriptome, immunome, and metabolome were systemically analyzed using the external TCGA dataset for validation. Kaplan-Meier and ROC curve analyses were performed to estimate the predictive capacity of M1-macrophage infiltration. Chi-square/Spearman/Mann Whitney U test are used to determine its correlation to genetic, biochemical, and clinicopathological parameters.

**Results:** M1 frequency is a robust biomarker for predicting the prognosis and response to ICBs, which is non-inferior to tumor mutation burden (TMB) or tumor neoantigen burden (TNB), and exceeds CD8 T cells, T cell inflamed gene expression profile (GEP), and PD-L1 expression. Moreover, M1 infiltration is associated with immune phenotypes (AUC = 0.785) and is negatively correlated with immune exclusion. Additionally, transcriptomic analysis showed immune activation in the high-M1 subgroup, whereas it showed steroid and drug metabolism reprograming in the M1-deficient subset, which characterized the limited sensitivity to ICB therapy. Notably, investigation of the corresponding intrinsic genomic profiles highlighted the significance of *TP53* and *FGFR* alterations.

**Conclusions:** M1 infiltration is a robust biomarker for immunotherapeutic response and immunophenotype determination in an mUC setting. Innate immunity activation involving macrophage polarization remodeling and anti-*FGFR* mutations may be promising strategies for synergy with anti-PD-L1 treatments and may help prolong the clinical survival of patients with mUC.

## Introduction

Advances in immunotherapy over the past few decades have revolutionized the clinical treatment landscapes of metastatic urothelial cancer (mUC) 1, 2, with six novel Food and Drug Administration approved agents, five immune checkpoint blockades (ICBs), and one *FGFR*-targeted agent 3, 4. While chemotherapy remains the routine clinical treatment, second-line therapy has shifted from single-agent chemotherapy to ICBs, owing to their durable response in a certain fraction of patients and their manageable safety profile 3, which was confirmed in a multicenter phase 3 randomized controlled trial (IMvigor211) 5. Additionally, ICBs have been adopted in the first-front treatment for patients with mUC in PD-L1-positive and platinum-ineligible settings 3.

Nevertheless, the beneficial and durable responses only occurred in a proportion of patients with mUC. Thus, applicable and reliable biomarkers to assess ICB therapeutic sensitivity are urgently warranted. To elucidate the underlying determinants of response and resistance, previous efforts have explored corresponding biomarkers, including PD-L1 expression levels 6, 7, tumor microenvironment gene signatures 8, 9, tumor mutation burden (TMB) 7, 8, T cell inflamed gene expression profile (GEP) 10, molecular subtypes 11, transforming growth factor β (TGFβ) signaling in fibroblasts 8, and fibroblast growth factor receptor 3 (*FGFR3*) alterations 12. Intriguingly, a recent study found *FGFR3* mutation status is not a biomarker of resistance to ICBs, despite its significant association with T-cell exclusion 13. Moreover, biomarkers for ICBs also interact with each other. For instance, high PD-L1 and CD8 expression had a significantly higher TMB or neoantigens in bladder urothelial carcinoma 14. Ongoing endeavors to investigate predictors of ICB therapeutic response shed new light on the complexity and significant role of tumor microenvironment (TME) 15-17. Apart from T cells, other infiltrating immune cells, such as neutrophils, natural killer cells, and macrophages are also potential candidates for cancer treatment response in several malignancies 18-20.

Preclinical research of TME has indicated the dual disparate role macrophages play in anti-neoplasia effect and in response to immunotherapy in various advanced-stage cancers 21, 22. Distinct macrophage profiles may exert diverse implications in the prediction of ICB sensitivity in advanced malignancies. Additionally, previous studies have also revealed metabolic pathways reprograming macrophage polarization (M1/M2) 23. Conversely, Anti-PD-L1 treatment also functionally remodels the macrophage compartment 24. TGF-β inhibition, combined with cytotoxic nanomedicine significantly improved immunostimulatory M1 macrophage content and boosted the efficacy of ICBs in breast cancer 25. However, translations of these preclinical investigations into clinical utility, and the functions that macrophages exert in mUC, have yet to be addressed. Here, by analyzing 348 patients with mUC treated with anti-PD-L1, we highlighted the robust predictive capacity of M1-infiltrating level in selecting patients that favorably respond to Atezolizumab and verified its crucial role in immunophenotype determination. Moreover, the corresponding immunome, transcriptome, genome, and metabolome are comprehensively discussed. We observed upregulated immune activation pathways in the high-M1 subset which identified favorable response to ICBs agents. In the low-M1 subset, we detected elevated expression of steroid metabolic and drug metabolic pathways, which characterize a poor immunotherapeutic sensitivity.

## Methods

### Data source and preprocessing

Genomic, transcriptomic, and matched clinical data from patients with metastatic urothelial cancer treated with an anti-PD-L1 agent (atezolizumab) 8 is available under the Creative Commons 3.0 license and can be downloaded from http://research-pub.gene.com/IMvigor210CoreBiologies. Data from The Cancer Genome Atlas (TCGA) were downloaded from the TCGA data portal (https://portal.gdc.cancer.gov/) in April 2019. RNA-seq count data were transformed into Transcripts Per Million (TPM) 26 to calculate gene signature scores. Updated clinical and pathological information for TCGA samples were obtained from GDC, using the R package TCGAbiolinks 27. Genomic data were analyzed using R (version 3.5.0) and R Bioconductor packages. Associated accessible codes of current work were merged into an R repository that is available at https://github.com/DongqiangZeng0808/mUC-M1.

### Genomic and clinical data sets with immune-checkpoint blockade

Five genomic and transcriptomic data sets from patients with metastatic urothelial cancer treated with an anti-PD-L1 agent (atezolizumab) 8, patients with metastatic melanoma and non-small-cell lung cancer treated with MAGE-3 agent-based immunotherapy 28, patients with advanced melanoma treated with various types of immunotherapy 29, a mouse model treated with anti-CTLA-4 from TCGA-SKCM cohort 30, and patients with metastatic gastric cancer treated with PD-1 inhibition (pembrolizumab) 10 were downloaded and analyzed to determine the predictive capacity of M1 macrophage and its comparison to its counterparts.

### Inference of immune cell infiltration and signature score

We integrated several computational tools 31-35 (**Supplementary Methods**) to estimate immune infiltration in the IMvigor210 and TCGA RNA-seq cohorts. Using the gsva algorithm, GO 36, KEGG 37, REACTOME 38, and HALLMARK 39 gene sets were employed to estimate pathway enrichment scores for each sample. Other prevalent gene signature scores with respect to tumor microenvironment, tumor intrinsic pathway, and metabolism were calculated for each sample using the PCA algorithm 9, 39 (see the detailed procedure in the **Supplementary Methods**).

### Lasso Cox model construction

The samples treated with atezolizumab in the IMvigor210 cohort were randomly separated into training/validation (6:4) sets for identifying and evaluating the predictors (see detailed patient characteristics in **Table S1**). All variables, including binary cell fractions and signature scores, were calculated separately using individual methods. The **Supplementary Methods** comprise all the methods used. Thereafter, these 7556 acquired features were merged into the feature matrix. A flow chart of the training lasso cox model was applied to depict the workflow of this study (**Figure S1**). Feature engineering was conducted to filter response-irrelevant and outcome-unrelated variables (see detailed patient features in **Table S2**). The penalized Cox regression model with LASSO penalty was applied to select the most powerful combination of prognostic markers based on 786 gene signatures after the procedure of feature engineering (**Supplementary Methods**). The optimal values of the penalty parameter lambda were determined through 10-times cross-validations 40. We then constructed a risk score model based on the level of the selected signatures using Cox regression coefficients in the training cohort. The same coefficients of each parameter were used to calculate the risk score in the validation set. This study was conducted and reported in line with the Transparent Reporting of a multivariate prediction model for Individual Prediction or Diagnosis (TRIPOD) guidelines 41.

### Differentially expressed gene (DEGs) analysis

All differential gene analyses were conducted using the DESeq2 package 42. Differential expressed gene analysis was performed using a generalized linear model with the Wald statistical test, with the assumption that the underlying gene expression count data were distributed per a negative binomial distribution with DESeq2. DEGs were considered for further analysis, with an adjusted *p*-value < 0.05. The adjusted *p*-value for multiple testing was calculated using the Benjamini-Hochberg correction 43.

### Functional and pathway enrichment analyses

Gene annotation enrichment analysis was performed using the R package clusterProfiler 44. Gene Ontology (GO) 36 and Kyoto Encyclopedia of Genes and Genomes (KEGG) 37 terms were identified with a strict cutoff of *p* < 0.01 and a false discovery rate (FDR) lower than 0.05. We also identified pathways that were up- and downregulated among groups by running a gene set enrichment analysis (GSEA) 45 of the adjusted expression data for all transcripts (**Supplementary Methods**).

### Statistics

The normality of the variables was tested using the Shapiro-Wilk normality test 46. For comparisons of two groups, statistical significance for normally distributed variables was estimated using unpaired Student's *t-*test, and non-normally distributed variables were analyzed using the Mann-Whitney *U* test. For comparisons of more than two groups, Kruskal-Wallis and one-way ANOVA tests were used for non-parametric and parametric methods, respectively 47. The correlation coefficient was computed using Spearman and distance correlation analyses. Two-sided Fisher's exact tests were used to analyze contingency tables. The cutoff values of each dataset were evaluated based on the association between survival outcome and signature score in each separate dataset using the survminer package. The Kaplan-Meier method was used to generate survival curves for the subgroups in each data set, and the log-rank (Mantel-Cox) test was used to determine statistically significant differences. The hazard ratios for univariate analyses were calculated using the univariate Cox proportional hazards regression model. The multivariate Cox regression model was used to determine independent prognostic factors. All statistical analyses were conducted using R (https://www.r-project.org/), and the *p*-values were two-sided. *p* values lower than 0.05 were considered statistically significant.

## Results

### M1 macrophage holds promise in predicting therapeutic response to PD-L1 blockade

**Figure 1:**
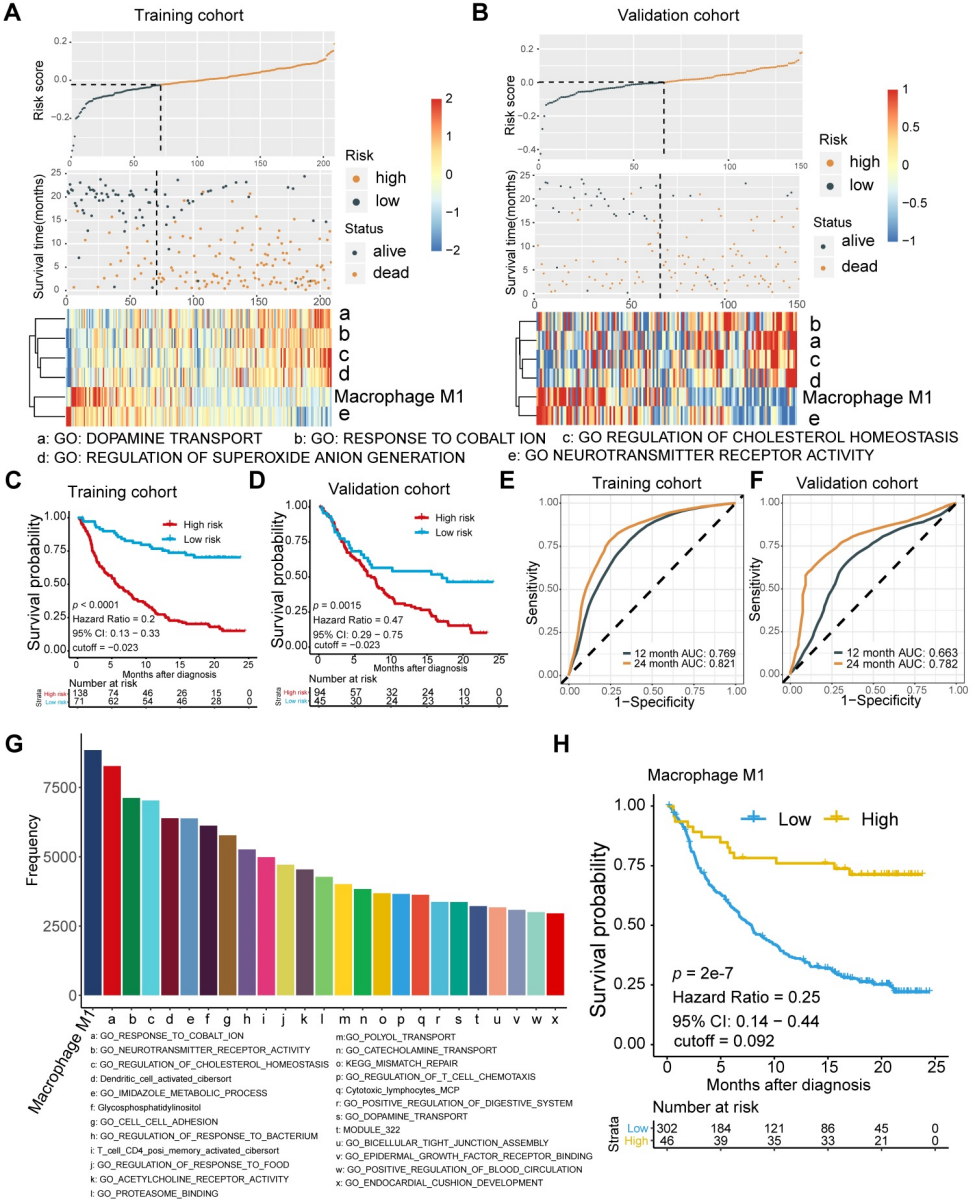
** Signature profile of training and validation cohort highlights M1 macrophage as latent biomarker. (A-B)** Establishment of a prognosis-predictive model dividing patients into high and low risk groups (**A**) and its validation (**B**). M1 macrophages and other metabolic pathways crucially contributed to the model were shown. Risk score and cutoff value on the top; Survival statues on the Middle; Gene expression heatmap (red: high expression; blue: low expression) of varying signatures selected by Lasso algorithm on the bottom. Feature selection processes and Feature matrix used to develop lasso model are displayed in Figure S1 and Table S2 and S3. **(C-D)** Risk score derived from the constructed model was significantly correlated with overall survival (Kaplan-Meier survival analyses, Training cohort: *p* < 0.0001, Hazard Ratio = 0.2, 95% CI: 0.13 - 0.33; Validation cohort: *p* < 0.0001, Hazard Ratio = 0.42, 95% CI: 0.27 - 0.63). **(E-F)** The established model held promise in predicting 12-month and 24-month survival. (Training cohort: 12-month AUC = 0.769, 24-month AUC = 0.821; Validation cohort: 12-month AUC = 0.663, 24-month AUC = 0.782). **(G)** Frequency of gene signatures selected by Lasso-bootstrapping highlighted the prominence of M1 macrophage contributing to predictive model. **(H)** M1 microphage infiltration was positively correlated with overall survival (*p* = 2e-7, Hazard Ratio = 0.25, 95% CI: 0.14 - 0.44).

To determine the optimal biomarkers for predicting ICB therapeutic sensitivity and to identify latent predictors, we explored the gene expression profile of 348 samples from Phase II Clinical Trial with signature deconvolution and developed a predictive model (**Figure 1A, Figure S1**;**Table S2** and** S3**). The constructed risk score model of the training cohort demonstrated that M1 macrophage and metabolic pathways, including cholesterol homeostasis, played significant roles in clustering the high-risk group and the low-risk group, which corroborated the validation cohort (**Figure 1A**). Analysis of the validation cohort corroborated these findings (**Figure 1B**). Kaplan-Meier survival analysis confirmed the survival discrepancy between the two risk groups (Training cohort: *p* < 0.0001, Hazard Ratio = 0.2, 95% CI: 0.13 - 0.33; Validation cohort: *p* = 0.0015, Hazard Ratio = 0.47, 95% CI: 0.29 - 0.75;** Figure 1C-D**), and the results of the ROC curve analysis validated the predictive value of the established risk model (**Figure 1E-F**).

Notably, subsequent bootstrapping indicated M1 macrophage as a promising biomarker surpassing more-than-7500 counterparts covering the TME, metabolic pathways, tumor intrinsic pathways, and hallmarks of cancer (**Figure 1G**). Kaplan-Meier survival analysis verified that patients with higher pretreatment M1 infiltrating frequency exhibited longer overall survival (M1: *p* = 2e-7, Hazard Ratio = 0.25, 95% CI: 0.14 - 0.44; **Figure 1H**; **Table S1**).

### Predictive robustness of M1 macrophage is non-inferior to that of TMB and TNB but superior to CD8+ T cell and PD-L1 expression

**Figure 2:**
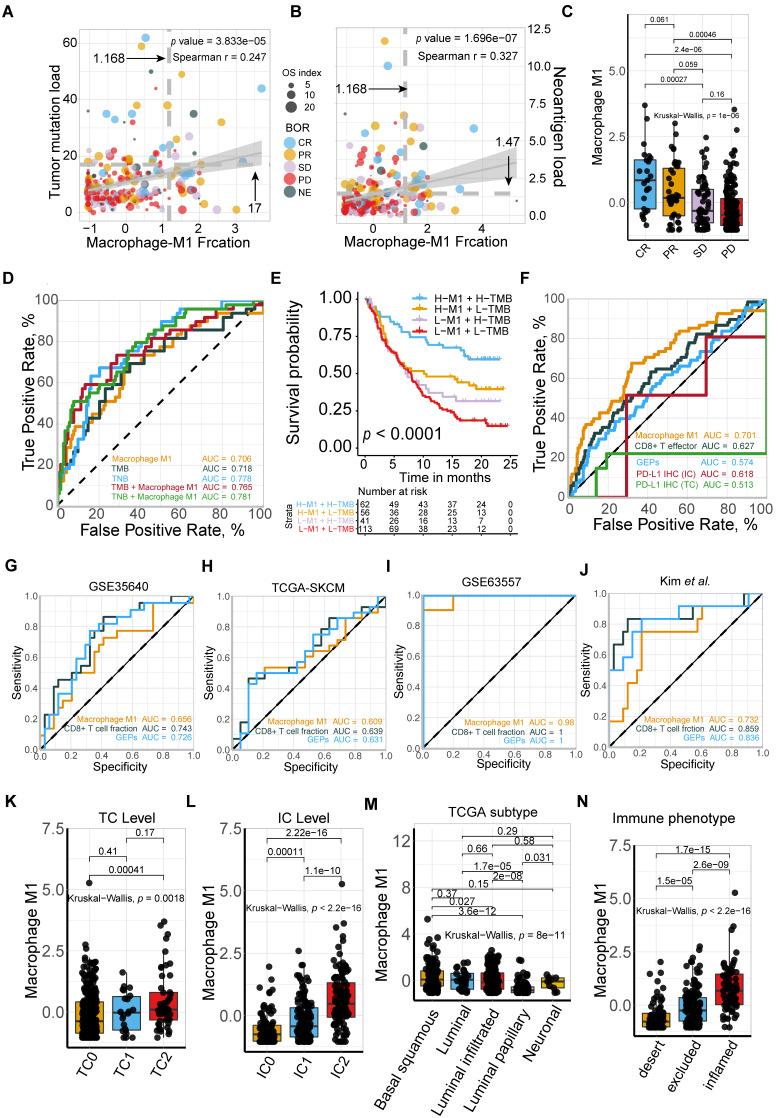
** Comparison and associations of published predictors with M1 macrophage. (A-B)** Modest but significant correlations of M1 macrophage were shown with TMB (**A**) and TNB (**B**) (Spearman, TMB: *p* = 3.833e-05, r = 0.247; TNB: *p* = 1.696e-07, r = 0.327). TMB: tumor mutation burden; TNB: tumor neoantigen burden. **(C)** M1-macrophage density is positively associated with response (Kruskal Wallis test, *p* = 1e-06), with association driven by the complete response group (CR versus PR, *p* = 6.1e-02; CR versus SD, *p* = 2.7e-04; CR versus PD, *p* = 2.4e-06). PD, progressive disease; SD, stable disease; PR, partial response. **(D)** Predictive value of M1 macrophage was comparable to TMB and TNB and elevated when combined with either TMB or TNB (M1: AUC = 0.706; TMB: AUC = 0.718; TNB: AUC = 0.778; M1+TMB: AUC = 0.765; M1+TNB: AUC = 0.781; M1: TMB: *p* = 0.811; M1: TNB: *p* = 0.134; M1: M1+TMB: *p* = 1.2e-02; M1: M1+TMB: *p* = 1e-04; M1+TMB: M1+TNB:* p* = 0.438). **(E)** M1 macrophage was statistically associated with favorable survival outcome in either high or low TMB subset (Kaplan-Meier survival analyses, *p* < 0.0001). A similar plot for tumor neoantigen burden is shown in Figure S2D. **(F)** Predictive efficacy of M1 macrophage were superior to CD8+T effector, T cell inflamed GEP, PD-L1 expression levels (TC/IC). PD-L1 expression on immune cells (IC) and tumor cells (TC) are assessed by SP142 immunohistochemistry assay and scored as IC0 (< 1%), IC1 (≥ 1% and < 5%), or IC2 (≥ 5%). (M1: AUC = 0.701; CD8: AUC = 0.627; IC: AUC = 0.618; TC: AUC = 0.513; M1: CD8: *p* = 3e-03; M1: GEP: *p* = 1.1e-05; M1: IC: *p* = 1.1e-02; M1:TC: *p* = 9.5e-06). **(G-J)** Predictive capacity of M1 macrophage was supported but not exceeding CD8+T effector and T cell inflamed GEP in various malignances cohorts under immunotherapy setting. (GSE35640: M1: AUC = 0.656; CD8+ T effector: AUC = 0.743; GEP: AUC = 0.726, M1: CD8: p = 0.241; M1: GEP: p = 0.405; CD8: GEP: p = 0.463; **(G)**; TCGA-SKCM: M1: AUC = 0.609; CD8+ T effector: AUC = 0.639; GEP: AUC = 0.63; M1: CD8: p = 0.681; M1: GEP: p = 0.800; CD8: GEP: p = 0.822; **(H)**; GSE63557: M1: AUC = 0.0.98; CD8 T effector: AUC = 1; GEP: AUC = 1; M1: CD8: p = 0.954; M1: GEP: p = 0.921; CD8: GEP: p = 0.956; **(I)**; Kim et al: M1: AUC = 0.732; CD8 T effector: AUC = 0.859; GEP: AUC = 0.836; M1: CD8: p = 0.048; M1: GEP: p = 0.077; CD8: GEP: p = 0.469; **(J)**. **(K-L)** PD-L1 expression, both TC (**K**) and IC (**L**), are associated with M1-macrophage infiltration (Kruskal Wallis test, *p* = 1.8e-3, *p* < 2.2e-16, respectively). IC0 tumors had a significantly lower M1-macrophage infiltration (*p* = 4.1e-04, *p* < 2.2e-16, respectively). Tumor tissue samples were scored through immunohistochemistry (IHC) for PDL1 expression on tumour-infiltrating immune cells (IC), which included macrophages, dendritic cells and lymphocytes. Specimens were scored as IHC IC0, IC1, IC2, or IC3 if <1%, ≥1% but <5%, ≥5% but <10%, or ≥10% of IC were PD-L1 positive, respectively. An exploratory analysis of PD-L1 expression on tumour cells (TC) was conducted. Specimens were scored as IHC TC0, TC1, TC2, or TC3 if <1%, ≥1% but <5%, ≥5% but <50%, or ≥50% of TC were PD-L1 positive, respectively. **(M)** Distribution of M1 macrophages varied between TCGA subtypes (Kruskal Wallis test, *p* = 8e-11). A similar plot for other molecular subtype classifications are shown in Figure S2F-G. **(N)** M1 macrophages predominantly enrich in inflamed immune subtypes defined by computational tools as well as IHC modality (Kruskal Wallis test, *p* < 2.2e-16). “Desert”: the prevalence of CD8+ cells was low (< 10 CD8+ cells in an area of tumour and tumour-associated stroma at a magnification of 200×; in larger specimens, this was calculated as the average of 10 representative fields of view). “Excluded”: CD8+ cells were exclusively seen in stroma immediately adjacent to or within the main tumour mass. “Inflamed”: CD8+ cells were seen in direct contact with malignant epithelial cells either in the form of spilling over of stromal infiltrates into tumour cell aggregates or of diffuse infiltration of CD8+ cells in aggregates or sheets of tumour cells.

Current tissue-based biomarkers for anti-PD-L1 therapy commonly involve TMB, TNB, PD-L1 IHC, and T cell-inflamed GEP. The correlation of M1 macrophage with established modalities and robustness comparison requires further investigation (**Figure 2**). Significant association between M1 frequency and favorable response to Atezolizumab treatment were indicated, despite the significant but modest correlations between M1 macrophage, TMB, and tumor neoantigen burden (TNB), respectively (Kruskal Wallis; M1: *p* = 1e-6; **Figure 2A-C**). M1 infiltrating density displayed non-inferior predictive value for atezolizumab treatment, in comparison with TMB and TNB (M1: AUC = 0.706; TMB: AUC = 0.718; TNB: AUC = 0.778; M1: TMB: *p* = 0.811; M1: TNB: *p* = 0.134;** Figure 2D**; **Table S4**), and functioned similarly but slightly better in estimating the overall survival outcome (M1: 12-month AUC = 0.647; 24-month AUC = 0.707; TMB: 12-month AUC = 0.644; 24-month AUC = 0.666; TNB: 12-month AUC = 0.647; 24-month AUC = 0.704; **Figure S2A-C**). Intriguingly, combining infiltrating M1 elevated the predictive accuracy of TMB and TNB even more than either of them alone (M1+TMB: AUC = 0.765; M1+TNB: AUC = 0.781; M1: M1+TMB: *p* = 1.2e-02; M1: M1+TMB: *p* = 1e-04; M1+TMB: M1+TNB: *p* = 0.438; **Figure 2D**;**Table S4**) and facilitated survival assessment (Kaplan-Meier analysis, M1+TMB binary: *p* < 0.0001; M1+TNB binary: *p* < 0.0001; **Figure 2E**, **Figure S2D**). Moreover, its predictive capacity exceeded the CD8+T effector, T cell-inflamed GEP, and expression of PD-L1 (TC or IC) (M1: AUC = 0.701; CD8 T effector: AUC = 0.627; GEP: AUC = 0.574; IC: AUC = 0.618; TC: AUC = 0.513; M1: CD8: *p* = 3e-3; M1: GEP: *p* = 1.1e-05; M1: IC: *p* = 1.1e-2; M1:TC: *p* = 9.5e-6; **Figure 2F**;**Table S4**), which collectively indicated that future improvements in diagnostic accuracy are likely to be made by including M1-macrophage signatures estimation via a computational algorithm that embraced a more favorable unbiased efficiency than PD-L1 IHC and by developing multiplex approaches combined with established biomarkers, TMB or TNB. Intriguingly, further exploration of varied cancer settings, other than bladder cancer, also supported the favorable predictive value of M1, but negated its superiority over CD8+ T effector and GEP in patients with metastatic melanoma and non-small-cell lung cancer treated with MAGE-3 agent based immunotherapy 28 (GSE35640: M1: AUC = 0.656; CD8+ T effector: AUC = 0.743; GEP: AUC = 0.726, M1: CD8: *p* = 0.241; M1: GEP: *p* = 0.405; CD8: GEP: *p* = 0.463; **Figure 2G**), cutaneous melanoma adopted various types of immunotherapy 29 (TCGA-SKCM: M1: AUC = 0.609; CD8+ T effector: AUC = 0.639; GEP: AUC = 0.63; M1: CD8: *p* = 0.681; M1: GEP: *p* = 0.800; CD8: GEP: *p* = 0.822; **Figure 2H**), mouse model treated with anti-CTLA4 30 (GSE63557: M1: AUC = 0.0.98; CD8 T effector: AUC = 1; GEP: AUC = 1; M1: CD8: *p* = 0.954; M1: GEP: *p* = 0.921; CD8: GEP: *p* = 0.956; **Figure 2I**) and patients with metastatic gastric cancer cohort derived from Kim *et al.* 48 treated with PD-1 inhibition (pembrolizumab) (Kim *et al.*: M1: AUC = 0.732; CD8 T effector: AUC = 0.859; GEP: AUC = 0.836; M1: CD8: *p* = 0.048; M1: GEP: *p* = 0.077; CD8: GEP: *p* = 0.469; **Figure 2J).** Additionally, despite the limited interobserver consistency, PD-L1 IHC is still the most well-established biomarker for anti-PD-L1 therapy 49, and patients with higher PD-L1 expression levels, either IC or TC, were observed with higher M1 infiltration (Kruskal Wallis, TC: *p* = 1.8e-3; IC: *p* < 2.2e-16; **Figure 2K-L**).

Moreover, a previous study indicated that neuronal subtype (N = 8) had a considerably high objective response rate (complete response rate: 25%, partial response rate: 75%) 50. However, the low occurrence of neuronal subtypes and other prevalent molecular subtypes estimated by the BLCA subtyping package may cripple the predictive capacity 11, 51 (**Table S4**). Although previous studies have provided evidence that molecular subtypes impacted prognosis and therapeutic response of mUC patients 52-54, modest or bare statistical correlation between molecular subtypes and immunotherapeutic response, as well as a varied distribution of M1 levels among different subtype classifications were observed in the current study (**Figure 2M**, **Figure S2E-F**;**Table S5**). Multi-variate Cox regression also recognized infiltrating M1 macrophage as a prognostic factor that collaborated with TMB and TCGA subtypes (M1: *p* = 0.0006; TMB: *p* = 0.0004; **Figure S2G**; **Table S5**).

Collectively, M1 macrophage and its combination with TMB or TNB are promising candidates for predicting the response to ICBs among patients with mUC to fine-tune the immune response and therapeutic strategies. Notably, we found that it holds promise in identifying immune exclusion of mUC patients, due to its significant correlation with immune infiltration (Kruskal-Wallis, *p* < 2.2e-16, **Figure 2N**).

### M1 macrophage is correlated with immunophenotypes and TME landscapes

**Figure 3:**
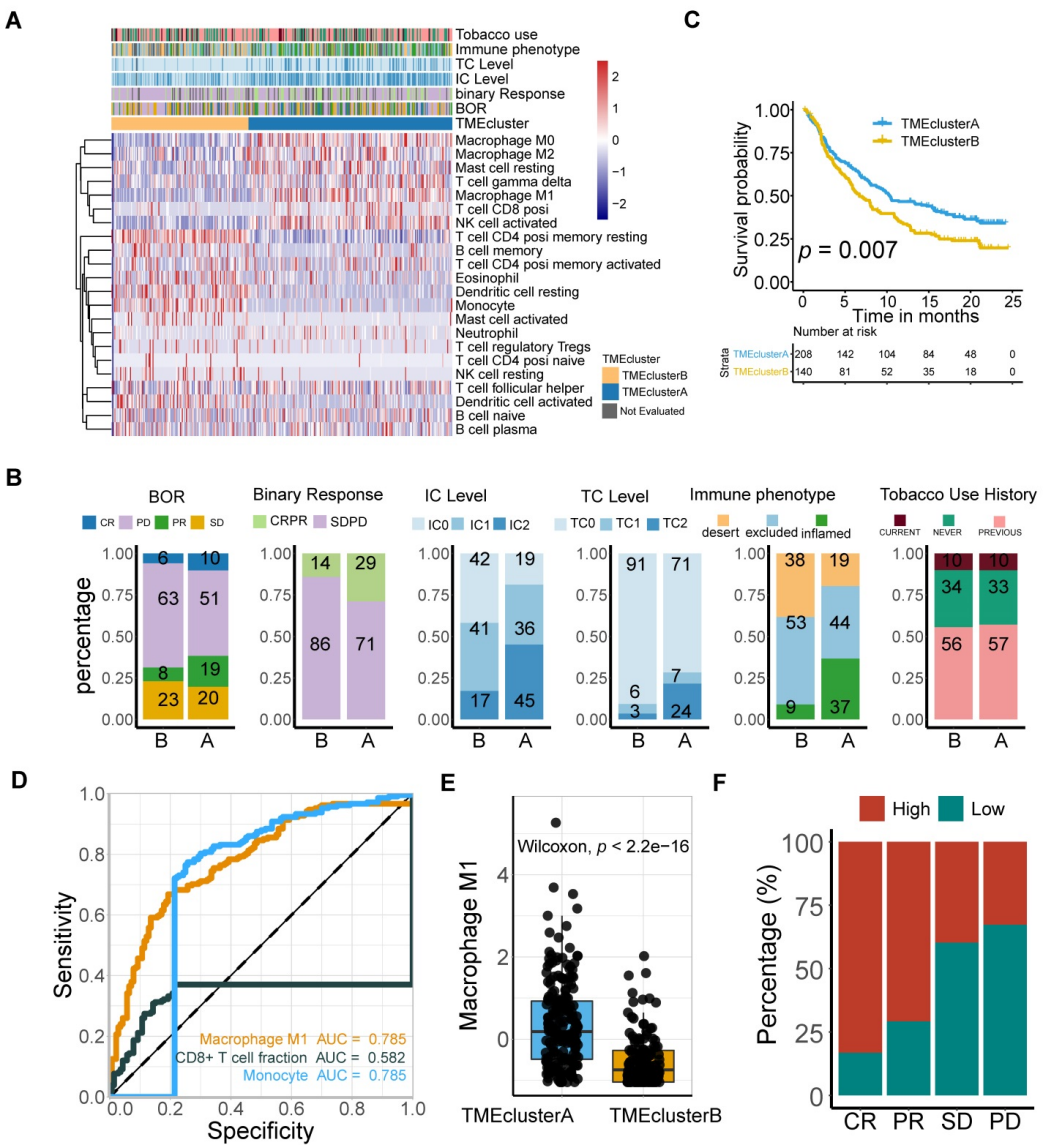
** M1-macrophage infiltration is related to immune phenotype. (A)** Heatmap of unsupervised consensus clustering based on the TME-cell signatures (red: high expression; blue: low expression) elucidated the TME pattern with two TME clusters A (blue) and B (yellow). Rows of the heatmap show expression of TME-infiltrating cell signatures (Z scores) calculated by CIBERSORT. Annotations are displayed on the top compressing tobacco use, immune phenotype, TC and IC levels, binary response and BOR. BOR: best overall response. **(B)** Distribution of clinicopathological parameters in TME cluster A and B with cluster A positively associated with favorable BOR and higher PD-L1 expression level (IC/TC) while negatively correlated with immune exclusion. Tobacco-use history allocated evenly. Additional clinical and molecular characteristics refer to Table S1. **(C)** TME clusters A statistically associated with better survival (Kaplan-Meier survival analysis, *p* = 7e-03). **(D)** Immunophenotype-determine capacity of M1 macrophage was similar to monocyte and exceeding CD8+T cell (M1: AUC = 0.785; monocyte: AUC = 0.785; CD8+T: AUC = 0.582). **(E-F)** TME cluster A was correlated with higher M1 macrophage (**E**) (Mann Whitney U test, *p* < 2.2e-16) which characterized favorable therapeutic response (**F**).

To further gain insight into the exact role M1 macrophage plays in determining the TME profile and immunophenotype, we performed unsupervised consensus clustering based on the TME-cell populations, developing a TME pattern with two clusters, TME cluster A and B (**Figure 3A**). A significantly better overall survival outcome was observed in the TME cluster A (*p* = 0.007; **Figure 3B**), which infiltrated with more M1 macrophages, resting mast cells, CD8 T cells, T cell gamma delta, and activated NK cells (**Figure 3A**).

Follow-up analyses of clinicopathological characteristics revealed that patients with a better best overall response (BOR), higher PD-L1 expression level, either on the surface of tumor cells (TC) or immune cells (IC), and inflamed phenotypes, were allocated more in TME cluster A (**Figure 3C**, **Table S1**), which was identified to have a lower immune exclusion, and therefore, a potentially better anti-tumor immune response. In addition, the influence of tobacco usage history on the TME pattern was excluded due to the balanced distribution (**Figure 3C**).

Given the immnunophenotype distribution discrepancy, we hypothesized that M1 macrophages hold promise in identifying immune phenotype of mUC, and subsequently validated its determining capacity comparable to monocytes and exceeding CD8 T cells (M1: AUC = 0.785; Mon: AUC = 0.785; CD8 T: AUC = 0.582; **Figure 3D**). The prolonged survival of TME cluster A (**Figure 3B**) was correlated with a higher M1-macrophage infiltration (Mann Whitney *U* test, *p* < 22e-16; **Figure 3E**) and a more favorable sensitivity to anti-PD-L1 therapy (**Figure 3F**), using the external TCGA dataset reproductively vouched for the aforementioned results (**Figure S3**).

Classic adaptive responses of macrophages include a wide spectrum of activation states compressing M1, M2, or M2-like. Prior researches have indicated the significance of myeloid intratumoral compartments and macrophage plasticity in immunotherapeutics 21, 55. In the current study, lower M2 frequency was statistically correlated with more favorable overall survival (**Figure S4A**, *p* = 0.011), despite its bare statistical correlations with M1 frequency, and immunotherapeutic responses (**Figure S4B-C**, *p* = 0.19), which suggested that M2 contributed to shaping the immune suppressive tumor microenvironment, as previously reported 21, 22*.* Furthermore, the M1/M2 ratio was significantly correlated with a better response to immune checkpoint blockade (**Figure S4D**, *p* = 4.6e-6). However, the M1/M2 ratio did not elevate the predictive capacity of M1 alone. In addition, M2 exerted inferior predictive sensitivity to anti-PD-L1 response (**Figure S4E**; **Table S4**; M1: AUC = 0.701; M2: 0.552; M1/M2 ratio: AUC = 0.653), which relies more on T cell activation and inflammatory microenvironment. In spite of the potential coexistence of M1 and M2-state, reeducating macrophages polarization toward M1-type state 22 may increase the survival rate among patients with mUC and collaborate with anti-PD-L1 therapy to advance patient response.

Overall, M1 macrophage infiltrating levels may also function as a robust indicator of immunophenotype that is significantly correlated with a favorable response. Therapy targeting macrophage remodeling may hold promise for synergy with ICBs and prolong the clinical survival of patients with mUC.

### High M1-macrophage infiltration suggests immune activation, while low infiltration indicates activated steroid and xenobiotic metabolism

**Figure 4:**
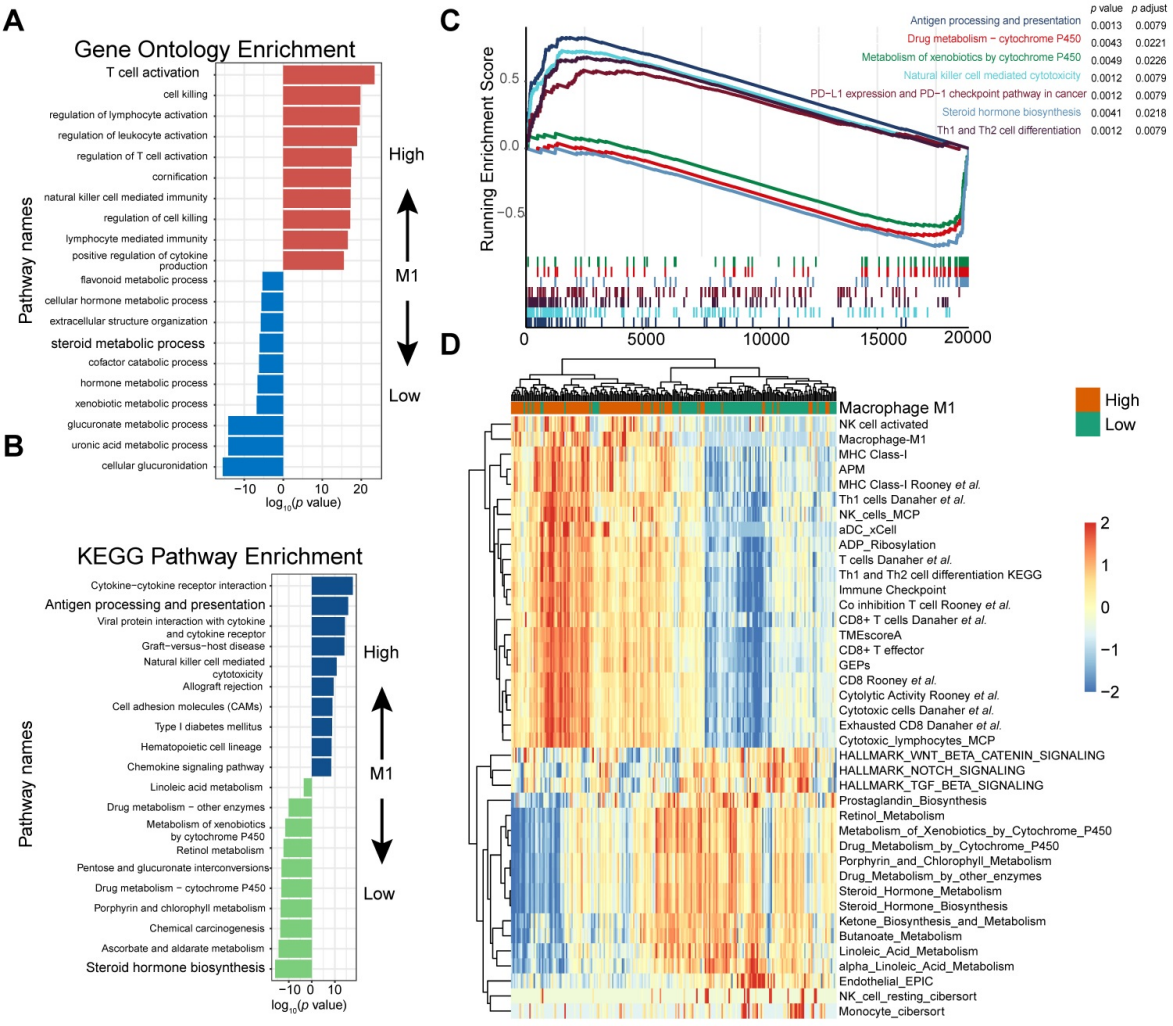
** M1 infiltration is correlated with distinct transcriptomic and metabolic traits. (A-B)** Gene ontology (GO) (**A**) and KEGG pathways (**B**) were significantly correlated with M1-macrophage infiltration with activation of steroid metabolism, xenobiotics metabolism in low-M1 subset and immune activation in high-M1 subset. The top ten genes per set are shown (ranked by single-gene *p* value, GO: red: high, blue: low; KEGG: blue: high, green: low); complete lists are given in Table S7. **(C)** GSEA analyses displayed key pathways enriched in high (up) and low (down) M1 subset. Gene sets that are inferred to reflect key underlying biological processes are coloured. (Green: metabolism of xenobolics by cytochrome P450; Scarlet: drug metabolism by cytochrome P450; Blue violet: steroid hormone biosynthesis; brick red: PD-L1 and PD-1 checkpoint pathway in cancer; Dark violet: Th1 and Th2 cell differentiation; Light blue: natural killer cell meditated cytotoxicity; Navy: antigen processing and presentation). Complete lists are given in Table S7. **(D)** Heatmap of unsupervised clustering different expressing gene signatures elucidated similar results (red: high expression; blue: low expression). Binary M1-macrophage infiltration was show as annotation on the top (red: high; green: low). Comprehensive signature information is displayed in Table S9.

To further identify the transcriptomic profiles in different M1 macrophage infiltrating settings and dissect the underlying mechanism contributing to its crucial predictive power in patients with mUC, 4,538 differentially expressed genes (DEGs) in the IMvigor210 cohort (**Table S6**), corresponding to pathway enrichments (**Figure 4A-C**; **Table S7** and** S8**) and gene signatures (**Figure 4D** and**Figure S5**;**Table S9**) were comprehensively explored.

Gene Ontology enrichment and KEGG enrichment analyses unanimously demonstrated that gene sets upregulated in higher M1 level subset were prevailingly enriched in immune activating process, which commonly indicated a better immunotherapeutic response, whereas those overexpressed in M1-deficient subset were enriched in steroid hormone metabolism, which could induce CD8+T cell exclusion in TME 56, xenobiotic metabolism, and drug metabolism, which may be collectively responsible for the dangerous insensitivity to immunotherapy (**Figure 4A-B**;**Table S7**). Gene set enrichment analysis (GSEA) results interpreted via hallmarks or KEGG database among all transcripts shared similar conclusions (**Figure 4C**;**Table S7**).

Consistently, heatmap suggested that high M1-infiltration closely correlated with gene signatures featuring immune activation, including overexpression of CD8 T effectors and interferons combined with other signatures characterizing favorable immunotherapy response in advanced cancers, such as expression of immune checkpoints and TME score 9 (**Figure 4D**). In contrast, patients with lower M1-infiltration are associated with endogenous metabolism activation, especially those involving steroids and drugs (**Figure 4D** and** S4**;**Table S9**). Comparable results were obtained via exploration of the TCGA dataset (**Figure S6** and** S7**;**Table S8** and** S9**). Taken together, the well-reproductive results intrigued the reasonable inference that immune activation in high M1-infiltration subsets and enhanced drug metabolism in the lower subset may intrinsically contribute to its robust predictive value to ICB therapeutic sensitivity.

### Tumor-intrinsic genomic alterations were related to the high M1 macrophage profile

**Figure 5:**
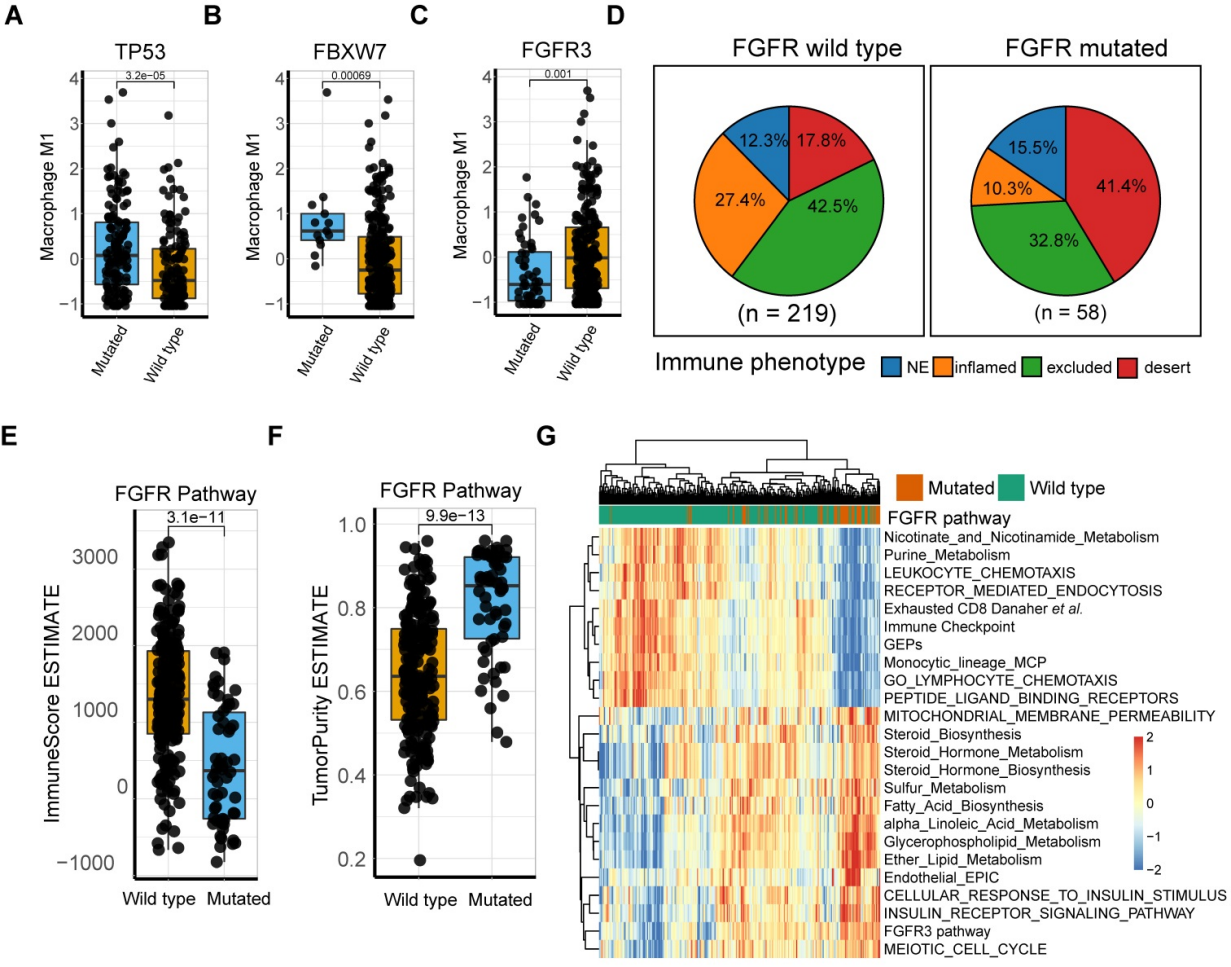
** M1 macrophage relevant intrinsic mutation profiles. (A-B)** *TP53* (**A**) and *FBXW7* (**B**) mutation were significantly associated with higher M1 infiltration compared with wild type (Mann Whitney U test, *p* = 3.2e-05,* p* = 6.9e-04, respectively). **(C)** Patients with *FGFR3* alteration, other than lack thereby, correlated with lower M1 infiltration (Mann Whitney U test, *p* = 1e-03). **(D)** Distribution of immune phenotypes in *FGFR* pathway mutation subset with more inflamed phenotype rate versus mutation deficiency. **(E-F)** *FGFR* pathway mutations were correlated with lower Immunoscore (Mann Whitney U test, *p* = 3.1e-11) and higher tumor purity (Mann Whitney U test, *p* = 9.9e-13) versus wild type. **(G)** Heatmap of different gene signatures expression profile via unsupervised clustering in *FGFR* mutated setting versus mutation deficiency. *FGFR* pathway mutation were associated more with steroid metabolisms while wild type with expression of immune cell signature and immune checkpoints. (red: high expression; blue: low expression; annotation: red: mutated; green: wild type).

Intensive investigation into the genetic profile demonstrated that M1-macrophage levels were significantly elevated in *FBXW7* and *TP53* mutation settings (Mann Whitney U test*, p* = 3.2e-5,* p* = 6.9e-4, respectively; **Figure 5A-B**;**Table S6**). *FBXW7* is a vital tumor suppressor and commonly deregulated ubiquitin-proteasome system protein in human cancer 57, which affects the tumor microenvironment and inhibits tumor metastasis by augmenting the activation of chemokine *CCL2* expression, which recruits monocytic myeloid-derived suppressor cells and macrophages to the tumor site 58. Additionally, the *TP53* gene suppresses tumorigenesis by determining DNA damage repair or apoptosis of damaged cells, promoting a tumor-inhibiting microenvironment through modulating macrophage polarization towards M1-state 59 and remodeling myeloid-T cell crosstalk 50. However, a decrease in M1-macrophage infiltration was observed in patients with *FGFR* mutation, compared with that in the wild type (Mann Whitney U test*, p* = 0.001; **Figure 5C**). Further analysis of the TCGA dataset externally supported the significance of these mutations (**Figure S8**). *FGFR* alterations have been recognized as a biomarker of resistance to ICBs 12 with anti-*FGFR* agents approved by the FDA 4. However, a recent study denied its response-predictive value, despite supporting its significant association with T-cell exclusion 13. In line with this, the current work demonstrated that *FGFR* mutated cases had a more deserted immune phenotype than the wild type (**Figure 5D**), as well as a lower Immunoscore and higher tumor purity (Mann Whitney U test*, p* = 3.1e-11,* p* = 9.9e-13, respectively; **Figure 5E-F**). Consistent with previous studies 60, *FGFR* pathway alteration was associated with alternative immune mechanisms, such as downregulated immune checkpoint pathways and elevated drug-resistance metabolism, especially steroid metabolism (**Figure 5G**, **S8A-B**), which collectively suggest that combination of *FGFR* inhibition and PD-L1 blockade may hold promise in elevating antitumor immunity*.* (**Figure 5G**, **S8A-B**; **Table S10-11**). However, a trend toward a better response to anti-PD-L1 therapy was observed in FGFR mutated patients, although statistical significance was not attained (**Figure S8 C-D;** *p* = 0.1399, *p* = 0.2691, respectively). Additionally, external validation of M1 macrophage related DEGs mutations (comprising *FGFR3*, *TP53*, and *FBXW7*) versus wild type in TCGA were also described (**Figure S8 E-G**).

## Discussion

Treatment with ICBs has revolutionized cancer therapy. To date, predictive biomarkers and strategies to augment clinical response to ICB therapy have largely focused on the T cell compartment 9, 10, 61, 62. However, other immune subsets and tumor intrinsic characteristics may also contribute to anti-tumor immunity or resistance to ICBs. Gene signatures derived from transcriptome hold great promise in personalized therapeutics 9, 10, 61-64. To gain insight into the mechanisms of the therapeutic response and resistance, 7,586 gene signatures (tumor and tumor microenvironment relevant), and infiltrated cell types were deconvoluted, followed by the integration of a machine learning algorithm 40 and a bootstrapping method. Our results suggest the promising value of M1 macrophage as a predictor of ICB therapy for mUC patients.

The comprehensive evaluation of genomic, transcriptomic, cellular, and molecular factors associated with response and resistance to anti-PD-L1 therapy in this large cohort of mUC patients revealed that M1 macrophage was not inferior to TMB, but was statistically superior to PD-L1 immunohistochemistry, CD8+ T cell signature 8, and T cell-inflamed GEP 10, 63. Recently, some clinical evidence indicated that tumor mutation burden has yet to prove its predictive or prognostic value for overall survival in order to become a reliable biomarker 65. Furthermore, some tumors with virus infection had a low mutation burden, but exhibited comparable immune infiltration, which subsequently facilitated patients to benefit from ICB immunotherapy 48, 66, suggesting a promising value of tumor microenvironment evaluation 9. As to PD-L1 IHC, several limitations were reported: multiple different assays are available, the scoring of immune cell PD-L1 expression by pathologists has poor interobserver reproducibility, and the PD-L1 expression is commonly reduced to a digital readout (+ vs -) without assessing its expression in the greater context of the TME 67. Discordances between assays and cutoff values based on tumor type of immunotherapy plague the biomarker field. With the utility of a computational algorithm 31, a standardized scoring system can be more efficient, unbiased, and cost effective. Molecular-subtype taxonomy has been widely investigated with respect to mUC, but with inconsistent results, as previously reported 8, 13. In another study 68, mUC patients with neuronal subtype (N = 8) had a considerably high objective response rate (complete response rate: 25%, partial response rate: 75%). However, few patients can be identified using this methodology, indicating that molecular subtypes are not capable of serving as a biomarker to guide immunotherapy. Our data also demonstrated that the distribution of M1 macrophages varied between molecular subtypes, but associations between molecular subtypes and treatment response were modest.

Notably, the combination of TMB or TNB with M1-macrophage signature evaluation statistically elevated the prognostic and predictive value of anti-PD-L1 efficacy, when compared with TMB, TNB, or M1 macrophages, indicating that there are some potential mechanisms beyond T cell anti-tumor immune processes mediated by mutations and tumor neoantigens. Our results provide evidence that M1 macrophages are predominantly enriched in inflamed immune subtypes defined using computational tools, as well as IHC modalities. Kim *et al*. corroborated these findings for triple-negative breast cancer and reported the same pretreatment trend in macrophage-enriched subtypes 23. Furthermore, DEGs, gene set enrichment analysis (GSEA), and gene set variation analysis (GSVA) demonstrated that tumors with M1-deficient subtype have dramatically higher activation in steroid metabolism, xenobiotics metabolism, and TGF-β signaling pathway, which were previously reported to develop immunosuppressive activity 8, 9, 55. The TCGA dataset of bladder cancer reproductively supported the aforementioned results. Taken together, the promising predictive value of M1 macrophage might contribute to a high accuracy in immune subtype prediction and potential interplay with stromal activation, exclusion of T cells, and tumor metabolism reprograming.

Macrophages are among the most abundant normal cells in the tumor microenvironment. A large number of preclinical studies 23, 69 revealed that macrophages undergo different activation and polarization processes: the classically activated subsets potentiate anti-tumor immunity, whereas the alternatively activated subsets promote tumor progression through multiple mechanisms. In line with our findings, Xiong *et al*. demonstrated that macrophage polarization was associated with the response of ICBs 24. As previously reported, simultaneous blocking of innate (CD47) and adaptive (PD-L1) checkpoints on tumor cells limits immune evasion and boosts anti-PD-L1 response 70, 71, suggesting that harnessing the innate immune system is a promising strategy to prolong survival outcomes of patients treated with ICB immunotherapy.

Our data revealed that a *TP53* mutation was associated with a more pro-inflammatory phenotype of macrophages in the IMvigor210 and TCGA cohorts. Conversely, the infiltration of M1 macrophages was markedly lower in tumors with *FGFR* pathway deficiency. Consistently with previous research 13, *FGFR* pathway mutations were not statistically correlated with anti-PD-L1 response, but were markedly enriched in desert-immune subtype and high tumor purity 72. The observation that *FGFR* pathway mutation is significantly associated with steroid metabolism activation, insulin receptor signaling pathway upregulation, cell cycle activation, and immune exclusion phenotype, inspired a rational hypothesis that anti-*FGFR* mutation may offer a way to tackle immune cell exclusion (including cytotoxic T cells and M1 macrophages) from the tumor center to boost tumor destruction via immunotherapy. Palakurthi *et al*. corroborated this theory for a non-small cell lung cancer mouse model, and reported the same immunological changes to support enhanced antitumor immunity 60.

Although the comprehensive evaluation of multi-omics data has yielded several important conclusions, the results of our study should be further validated in a prospective cohort of patients receiving immunotherapy to more precisely define the cutoff values for clinical application. The deficiency of this study is that the cutoff of M1 was derived from big dataset research, which is insufficient for clinical transformation, because the abundance of M1 was normalized within the dataset and cannot be evaluated individually. To tackle this challenge, we are working to generate a panel that can apply M1 signature genes and NanoString technology to estimate the fraction of M1 individually and normalize gene expression using housekeeping genes. Furthermore, our research observed a markedly close interaction between macrophages and anti-tumor immunity, such as cytotoxic T cells, as well as tumor intrinsic genomic alterations. The underlying mechanism of these observations should be systematically elucidated *in vitro* and *in vivo* to boost anti-tumor immunity optimally.

### Author contributions

Dongqiang Zeng, Zilan Ye and Wangjun Liao had full access to all the data in the study and take responsibility for the integrity of data and the accuracy of the data analysis.

*Study concept and design:* Dongqiang Zeng, WangJun Liao; *Acquisition of data:* Zilan Ye, Jianhua Wu, Jiani Wu, Gaofeng Wang; *Analysis and interpretation of data:* Dongqiang Zeng, Zilan Ye, Miaohong Wang; *Drafting of the manuscript:* Zilan Ye, Dongqiang Zeng, Rui Zhou, Yiqiang Huang*;Critical revision of the manuscript for important intellectual content:* Nailin Li, Yulin Liao, Jianping Bin, Min Shi, Wangjun Liao; *Statistical analysis:* Xinxiang Fan, Dongqiang Zeng, Zilan Ye; *Obtaining funding:* Wangjun Liao. *Administrative, technical, or material support:* Dongqiang Zeng, Huiying Sun; *Supervision:* Wangjun Liao; *Other:* None.

## Supplementary Material

Supplementary figures.

Supplementary tables.
