# Supplementary material for: Macrophage correlates with immunophenotype and predicts anti-PD-L1 response of urothelial cancer
Source: Theranostics. 2020 May 25;10(15):7002–14. doi: 10.7150/thno.46176 (PMC7295060; doi:10.7150/thno.46176)
Supplement: Supplementary file 1 — Supplementary figures. [file thnov10p7002s1.pdf]

Macrophage correlates with immunophenotype and predicts anti-PD-L1 response of urothelial cancer

Dongqiang Zeng[1†], Zilan Ye[1†], Jiani Wu[1], Rui Zhou[1], Xinxiang Fan[2], Gaofeng Wang[3], Yiqiang Huang[4], Jianhua Wu[1], Huiying Sun[1], Miaohong Wang[1], Jianping Bin[5], Yulin Liao[5], Nailin Li[6], Min Shi[1], Wangjun Liao[1*]

[1]Department of Oncology, Nanfang Hospital, Southern Medical University, Guangzhou, Guangdong, P. R. China; [2]Department of Urology, Sun Yat-sen Memorial Hospital, Sun Yat-sen University, Guangzhou, China; [3]Department of Dermatology, Johns Hopkins School of Medicine, Baltimore, Maryland, USA; [4]Department of Urology, Daping Hospital, Army Medical University, Chongqing, P.R. China; [5]Department of Cardiology, State Key Laboratory of Organ Failure Research, Nanfang Hospital, Southern Medical University, Guangzhou, China; [6]Karolinska Institutet Department of Medicine-Solna, Clinical Pharmacology Group, Karolinska University Hospital-Solna, 171 76, Stockholm, Sweden.

† These authors contributed equally to this work.
* **Correspondence author:** Department of Oncology, Nanfang Hospital, Southern Medical University, 1838 North Guangzhou Avenue, Guangzhou 510515, China.
E-mail: nfyyliaowj@163.com (W. Liao)

Supplementary Methods

1. Inference of Immune Cell Infiltration

- **CIBERSORT** [1]**:** To quantify the proportions of immune cells in the tumor samples, we used the CIBERSORT algorithm and the LM22 gene signature, which allows for highly sensitive and specific discrimination of 22 human immune cell phenotypes including B cells, T cells, NK cells, macrophages, dendritic cells (DCs), and myeloid subsets. CIBERSORT is a deconvolution algorithm that uses a set of reference gene expression values (a signature with 547 genes) considered a minimal representation for each cell type and, based on those values, infers cell type proportions in data from bulk tumor samples with mixed cell types using support vector regression. Gene expression profiles were prepared using standard annotation files and data were uploaded to the CIBERSORT web portal (http://cibersort.stanford.edu/), with the algorithm run using the LM22 signature at 1,000 permutations.
- **MCP-counter** [2]**:** Proportions of stromal cell were also applied Microenvironment Cell Populations-counter (MCP-counter) method, which allows the robust quantification of the absolute abundance of eight immune and two stromal cell populations in heterogeneous tissues from transcriptomic data.
- **TIMER** [3]**:** Allows explorations of the disease-specific clinical impact of different immune infiltrates in the tumor microenvironment. TIMER was developed to estimate the abundance of six tumor-infiltrating immune cell types (B cells, CD4 T cells, CD8 T cells, neutrophils, macrophages, and dendritic cells) to study 23 cancer types in The Cancer Genome Atlas (TCGA). This tool was validated thanks to Monte Carlo simulations, orthogonal estimates from DNA methylation-based inferences, as well as pathological assessment.
- **EPIC** [4]: EPIC package uses a constrained least square minimization to estimate the proportion of each cell type with a reference profile and another uncharacterized cell type in bulk gene expression samples.
- **xCell** [5]: Performs cell type enrichment analysis from gene expression data for 64 immune and stroma cell types. xCell is a gene signatures-based method learned from thousands of pure cell types from various sources. xCell applies a technique for reducing associations between closely related cell types. xCell signatures were validated using extensive in-silico simulations and cytometry immunophenotyping, and were shown to outperform previous methods.

2. Tumor Purity Assessment
Tumor purity was assessed computationally in all longitudinal samples using estimates derived from RNA-seq data using ESTIMATE algorithm [6] that uses gene expression signatures to infer the fraction of stromal and immune cells in tumor samples.

3. BLCA molecular subtyping
Molecular subtypes of tumor samples were estimated using the approach of BLCAsubtyping [7], an R package which integrates six published molecular classifications.

4. Gene expression profile obtained from GEO and preprocessing
Raw data from the microarray datasets generated using Affymetrix® and Illumina® were downloaded from the Gene Expression Omnibus (https://www.ncbi.nlm.nih.gov/geo/). The raw data for the dataset from Affymetrix® were processed using the RMA algorithm for background adjustment using the Affy package [8]. RMA was used to perform background adjustment, quantile normalization, and final summarization of oligonucleotides per transcript using the median polish algorithm. The raw data for the dataset from Illumina® were processed using the lumi package. The ComBat algorithm [9] was applied to reduce the likelihood of batch effects from non-biological technical biases.

5. Calculating signature score of gene sets using PCA algorithm
For gene expression (normalized by RMA or TPM methods) matrix, the expression of each gene in a signature was standardized so that its mean expression was zero, and standard deviation was 1 across samples. Then, a principal component analysis (PCA) was performed, and principal component 1 was extracted to serve as the gene signature score. This approach has the advantage of focusing the score on the set with the largest block of well-correlated (or anti-correlated) genes in the set, while down-weighting contributions from genes that do not track with other set members [10, 11].

6. Feature Engineering
To avoid the shortcoming of overfitting of lasso cox regression model developed by the training dataset, feature engineering was conducted. Firstly, all features (N = 7556) were standardized across all samples. Then, univariate Log-rank (Mantel-Cox) test was conducted to seek out signatures that were associated with anti-PD-L1 survival outcome with a cutoff of P = 0.01. Moreover, Mann-Whitney *U* test was applied to find out features related to treatment response (CR/PR vs SD/PD) with a cutoff of $p = 0.05$. As a result, we got 780 features for training a LASSO cox regression model.

7. Bootstrap sampling to obtain robustness of predictors

LASSO Cox regression [12] was performed for the signatures (N = 780) passing the initial filtering of feature engineering using 80 percent of the training set, randomly selected. This procedure was repeated 10,000 times and we got 10,000 cox regression model which was used to calculate the frequency of each feature that was enrolled in anti-PD-L1 immunotherapy predictive model.

8. Assessment of prognostic and predictive value of biomarkers

The sensitivity and specificity of the survival prediction based on the risk model and signature scores were depicted by the time-dependent receiver operating characteristic curve (ROC) and quantified by the area under the ROC (AUC) using timeROC and survivalROC package [13]. “roc.test” function of pROC package [14] was used to compares the AUC or partial AUC of two correlated or uncorrelated ROC curves.

9. Consensus clustering of TME infiltrating pattern

Cell fraction matrix deconvoluted by CIBERSORT [1] was scaled, and unsupervised clustering methods (K-means) [15] for analysis of dataset was applied to identify immune cell infiltrating pattern and classify patients for further analysis. This procedure using ConsensuClusterPlus R package [16] was repeated 1000 times to ensure the stability of classification.

10. Gene Set Enrichment Analysis and visualization

Gene sets were downloaded from the MSigDB database (v.6.2) of Broad Institute [17], then HALLMARK [18] and KEGG [19] gene sets were selected to perform over-representation hypergeometric test [17]. Enrichment *P* values were based on 1,000 permutations and subsequently adjusted for multiple testing using the Benjamini-Hochberg procedure to control the FDR [20]. The R package enrichplot (https://github.com/GuangchuangYu/enrichplot), implements several visualization methods to help interpreting enrichment results and was adopted to visualize GSEA result.

11. Other gene signatures enrolled in this study

To characterize the metabolism, immune microenvironment and other prevalent gene signatures activation in each tumor samples, PCA algorithm was apply to determine the pathway activity using gene sets (see **Supplementary Table S11**) curated by Mariathasan et al. [10], Cristescu et al. [21], Rooney et al. [22], Rosario et al. [23] and Zeng et al.[24]. We thereby obtained, for each signature, an enrichment score per sample that indicated the extent of upregulation or downregulation of the associated genes. A minimum overlap of two genes was required.

References

1. Newman AM, Liu CL, Green MR, Gentles AJ, Feng W, Xu Y, et al. Robust enumeration of cell subsets from tissue expression profiles. Nat Methods. 2015; 12: 453-7.
2. Becht E, Giraldo NA, Lacroix L, Buttard B, Elarouci N, Petitprez F, et al. Estimating the population abundance of tissue-infiltrating immune and stromal cell populations using gene expression. Genome Biol. 2016; 17: 218.
3. Li B, Severson E, Pignon JC, Zhao H, Li T, Novak J, et al. Comprehensive analyses of tumor immunity: implications for cancer immunotherapy. Genome Biol. 2016; 17: 174.
4. Racle J, de Jonge K, Baumgaertner P, Speiser DE, Gfeller D. Simultaneous enumeration of cancer and immune cell types from bulk tumor gene expression data. Elife. 2017; 6.
5. Aran D, Hu Z, Butte AJ. xCell: digitally portraying the tissue cellular heterogeneity landscape. Genome Biol. 2017; 18: 220.
6. Yoshihara K, Shahmoradgoli M, Martinez E, Vegesna R, Kim H, Torres-Garcia W, et al. Inferring tumour purity and stromal and immune cell admixture from expression data. Nat Commun. 2013; 4: 2612.
7. Kamoun A, de Reynies A, Allory Y, Sjodahl G, Robertson AG, Seiler R, et al. A Consensus Molecular Classification of Muscle-invasive Bladder Cancer. Eur Urol. 2019.
8. Gautier L, Cope L, Bolstad BM, Irizarry RA. affy--analysis of Affymetrix GeneChip data at the probe level. Bioinformatics. 2004; 20: 307-15.
9. Johnson WE, Li C, Rabinovic A. Adjusting batch effects in microarray expression data using empirical Bayes methods. Biostatistics. 2007; 8: 118-27.
10. Mariathasan S, Turley SJ, Nickles D, Castiglioni A, Yuen K, Wang Y, et al. TGFbeta attenuates tumour response to PD-L1 blockade by contributing to exclusion of T cells. Nature. 2018; 554: 544-8.
11. Zeng D, Li M, Zhou R, Zhang J, Sun H, Shi M, et al. Tumor Microenvironment Characterization in Gastric Cancer Identifies Prognostic and Immunotherapeutically Relevant Gene Signatures. Cancer Immunology Research. 2019; 7: 737-50.
12. Goeman JJ. L1 penalized estimation in the Cox proportional hazards model. Biom J. 2010; 52: 70-84.
13. Kamarudin AN, Cox T, Kolamunnage-Dona R. Time-dependent ROC curve analysis in medical research: current methods and applications. BMC medical research methodology. 2017; 17: 53.
14. Robin X, Turck N, Hainard A, Tiberti N, Lisacek F, Sanchez J-C, et al. pROC: an open-source package for R and S+ to analyze and compare ROC curves. BMC Bioinformatics. 2011; 12: 77.
15. Hartigan JA WM. Algorithm AS 136: A k-means clustering algorithm. . Appl Stat. 1979; 28.
16. Monti S, Tamayo P, Mesirov J, Golub T. Consensus Clustering: A Resampling-Based Method for Class Discovery and Visualization of Gene Expression Microarray Data. Machine Learning. 2003; 52: 91-118.
17. Subramanian A, Tamayo P, Mootha VK, Mukherjee S, Ebert BL, Gillette MA, et al. Gene set enrichment analysis: A knowledge-based approach for interpreting genome-wide expression profiles. Proceedings of the National Academy of Sciences. 2005; 102: 15545-50.
18. Liberzon A, Birger C, Thorvaldsdottir H, Ghandi M, Mesirov JP, Tamayo P. The Molecular Signatures Database (MSigDB) hallmark gene set collection. Cell Syst. 2015; 1: 417-25.
19. Tanabe M, Furumichi M, Kanehisa M, Kawashima M, Sato Y. KEGG as a reference resource for gene and protein annotation. Nucleic Acids Research. 2015; 44: D457-D62.
20. Benjamini Y, Hochberg Y. Controlling the False Discovery Rate: A Practical and Powerful Approach to Multiple Testing. Journal of the Royal Statistical Society: Series B (Methodological). 1995; 57: 289-300.
21. Cristescu R, Mogg R, Ayers M, Albright A, Murphy E, Yearley J, et al. Pan-tumor genomic biomarkers for PD-1 checkpoint blockade-based immunotherapy. Science. 2018; 362.
22. Rooney MS, Shukla SA, Wu CJ, Getz G, Hacohen N. Molecular and genetic properties of tumors associated with local immune cytolytic activity. Cell. 2015; 160: 48-61.
23. Rosario SR, Long MD, Affronti HC, Rowsam AM, Eng KH, Smiraglia DJ. Pan-cancer analysis of transcriptional metabolic dysregulation using The Cancer Genome Atlas. Nat Commun. 2018; 9: 5330.
24. Zeng D, Li M, Zhou R, Zhang J, Sun H, Shi M, et al. Tumor Microenvironment Characterization in Gastric Cancer Identifies Prognostic and Immunotherapeutically Relevant Gene Signatures. Cancer Immunology Research. 2019; 7: 737.

Supplementary Figures

Figure S1

Flowchart of LASSO cox regression model construction and validation.

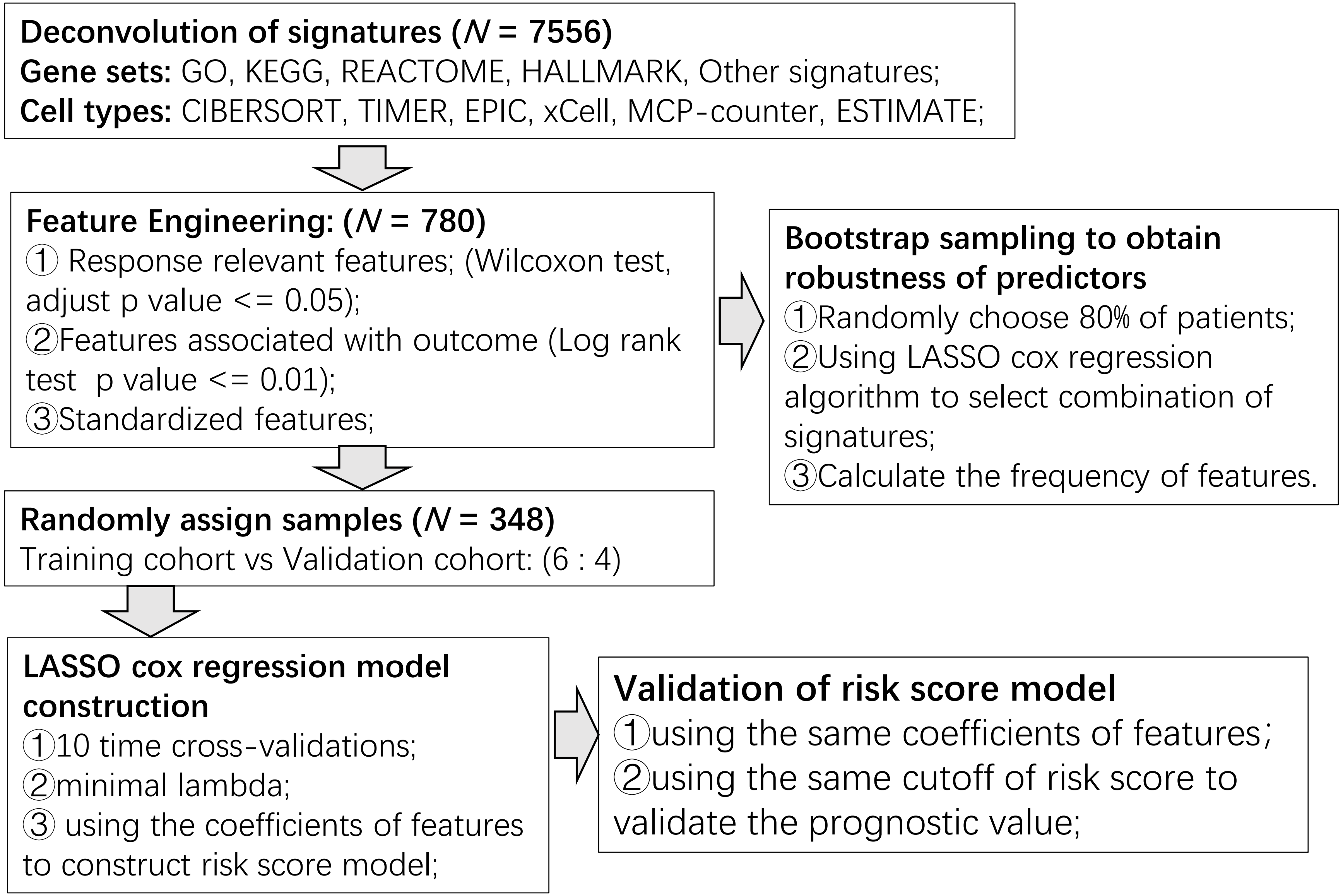

Figure S2

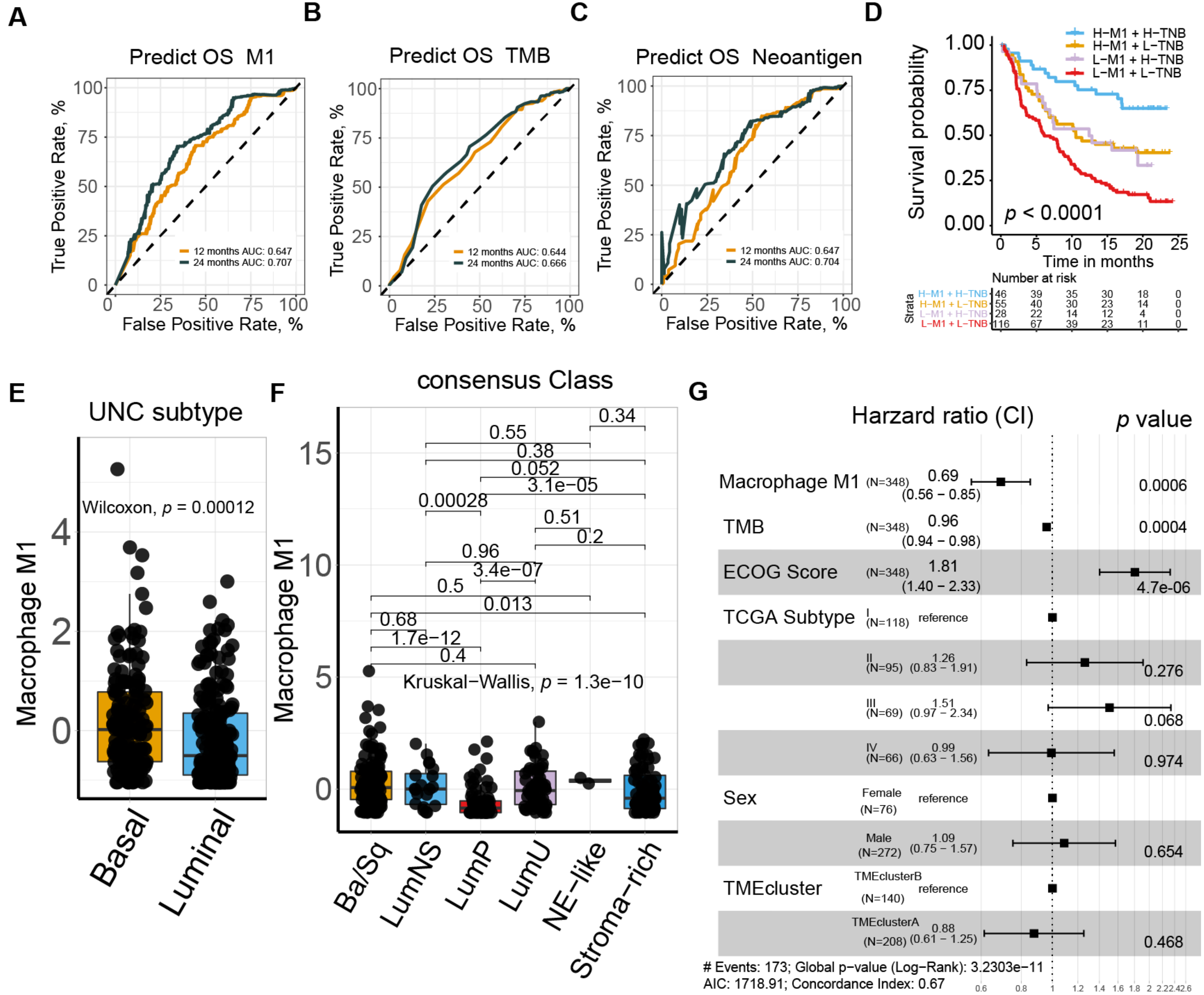

Correlation and comparison of M1 macrophage with reported biomarkers.
(A-C) The predictive capacity of M1 macrophage (**A**) in estimating overall survival was superior to TMB (**B**), TNB (**C**). (M1: 12-month AUC = 0.647, 24-month AUC = 0.707; TMB: 12-month AUC = 0.644, 24-month AUC = 0.666; TNB: 12-month AUC = 0.647, 24-month AUC = 0.704.) TMB: tumor mutation burden; TNB: tumor neoantigen burden.
(D) M1 macrophage was statistically associated with favorable survival outcome independent of TNB (Kaplan-Meier survival analyses, $p < 0.0001$).
(E-F) Distribution of M1 macrophage varied among UNC subtypes (**F**) and Consensus Class (**G**) respectively (Mann Whitney U test, p = 1.2e-04, p = 1.3e-10; respectively).
(G) Multi-variates regression analysis of hazard ratio among M1 macrophage and prior reported biomarkers. M1 macrophage collaborated with TMB were protect factors in resistance to tumor progression. Additional statistical analysis of biomarkers refer to Table S6.

Figure S3

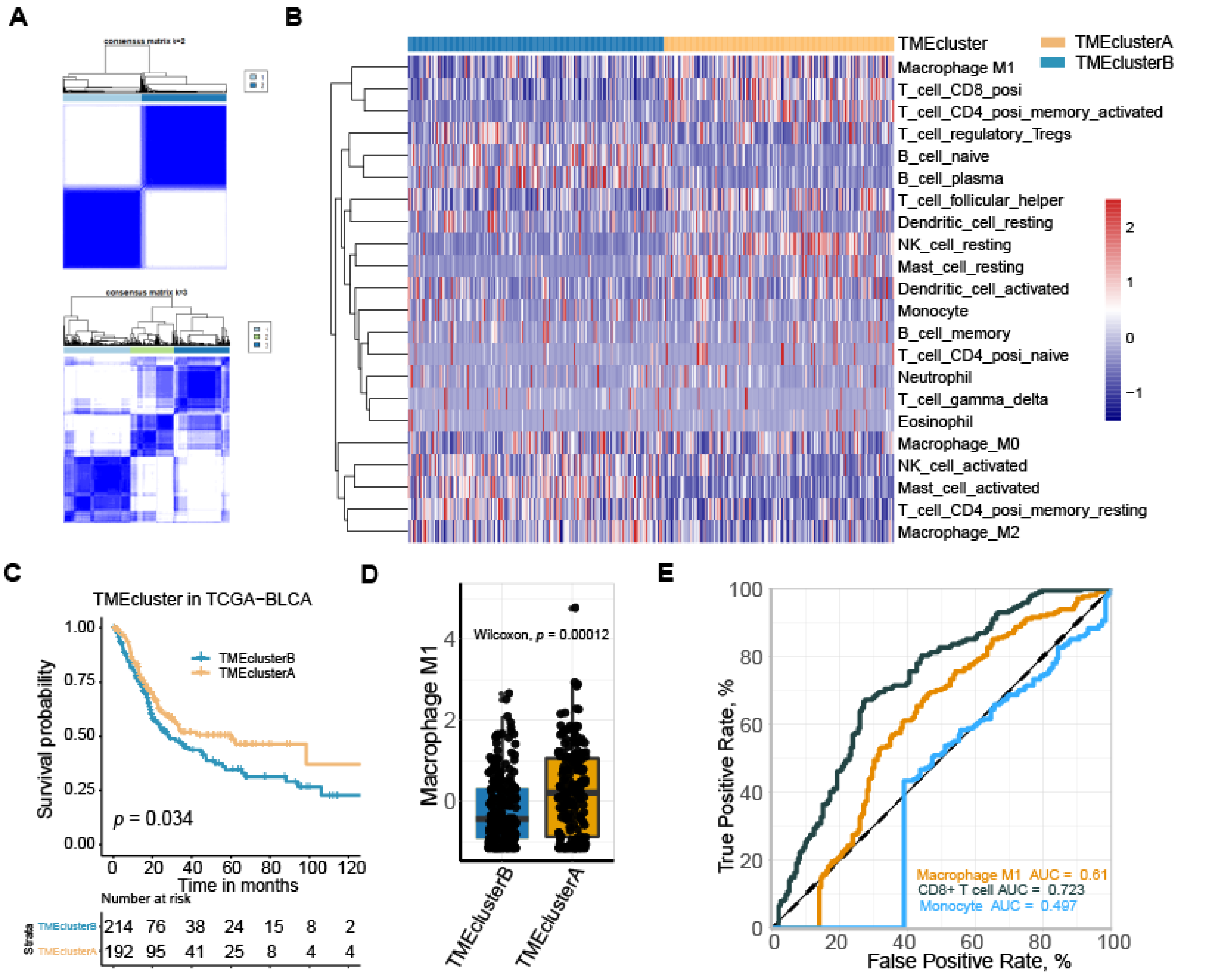

M1 macrophage reproductively correlates with immunophenotype in TCGA.

(A) Unsupervised consensus clustering analysis of TCGA based on the TME-cell signatures inclined to divide into two TME clusters. The plot of two and three clusters were both displayed.

(B) Heatmap of (red = high expression; blue = low expression) TME pattern of TCGA data with two TME clusters A (blue) and B (yellow). Rows of the heatmap show expression of TME-infiltrating cell signatures (Z scores) calculated by CIBERSORT.

(C) TME clusters A statistically associated with better survival (Kaplan-Meier survival analysis, p = 3.4e-02) in TCGA BLCA dataset.

(D) TCGA BLCA dataset validated the significant correlation between TME cluster A and high M1 infiltration (Mann Whitney U test, $p < 1.2\text{e-}04$)

(E) Immunophenotype-determine capacity of M1 macrophage was validated in analysis of TCGA (M1: AUC = 0.61; monocyte: AUC = 0.497; CD8+T: AUC = 0.723).

Figure S4

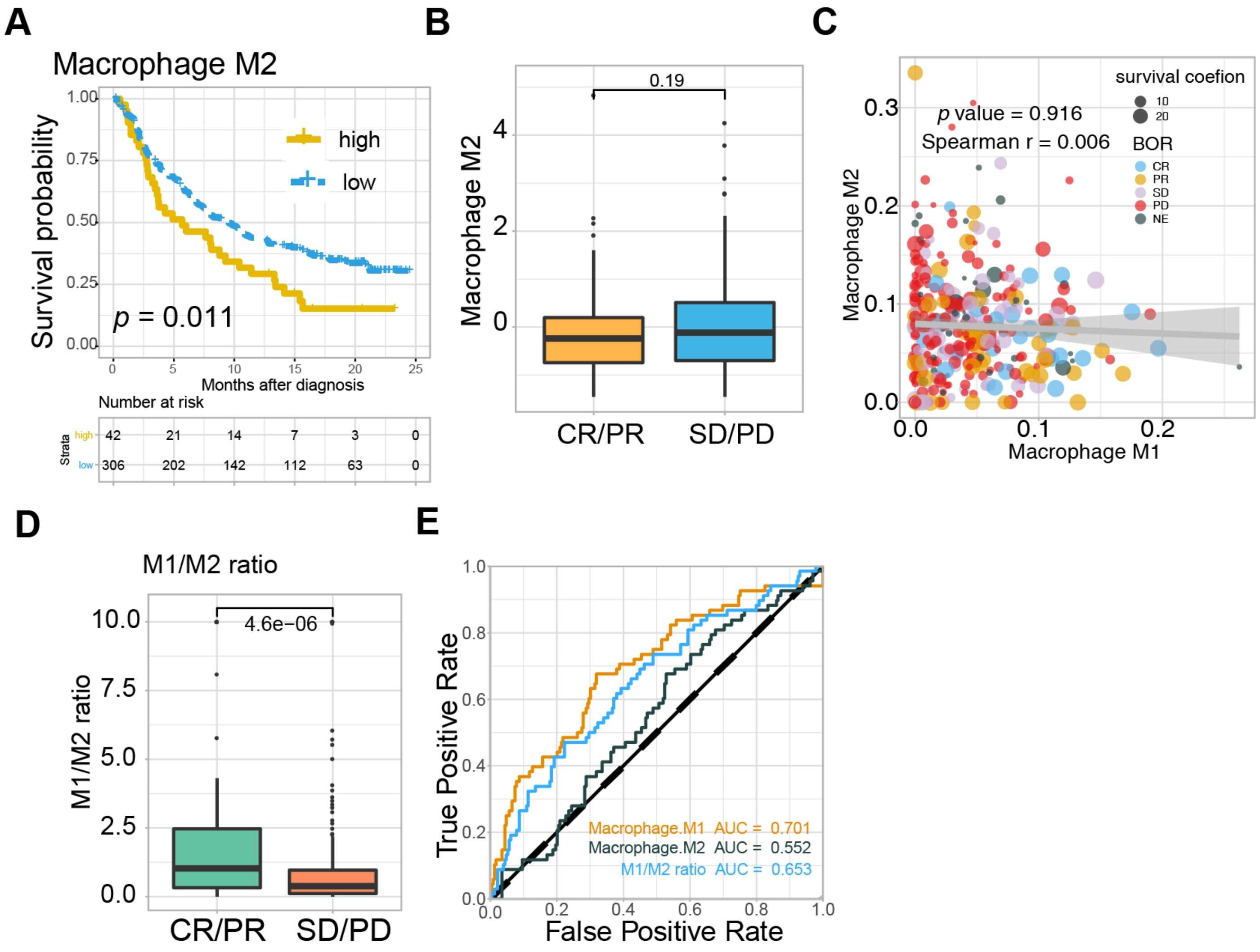

Combination with macrophage M2 did not raise the predictive value of predictive capacity of M1 alone .

(A) Lower M2 frequency was statistically correlated with more favorable overall survival (*p* = 0.011)

(B) M2 quantification discrepancy in different response subgroups didn't reach statistical significance (*p* = 0.19).

(C) Bare statistical correlation was observed between macrophage M1 and M2 frequency (*p* = 0.916; r = 0.006).

(D) High M1/M2 ratio was significantly associated with better response to anti-PD-L1 therapy (*p* = 4.6e-06).

(E) ROC curve suggested that M2 exerted inferior predictive sensitivity to anti-PD-L1 response. M1/M2 ratio didn't elevate the predictive capacity of M1 alone. (M1: AUC = 0.701; M2: 0.552; M1/M2 ratio: AUC = 0.653). Corresponding *p* values are exhibited in Table S4.

Figure S5

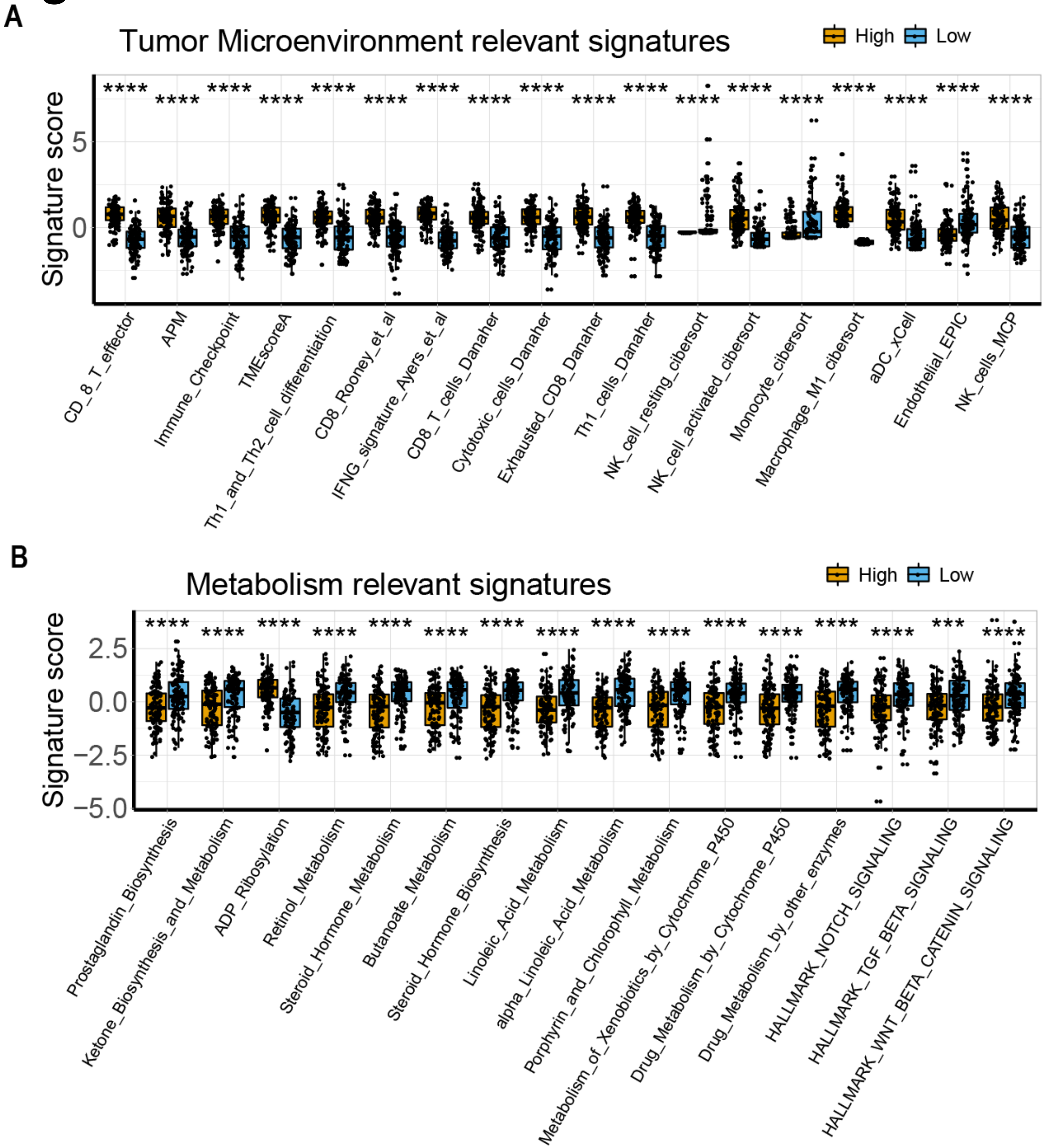

Tumor microenvironment and metabolic signatures expression in IMvigor210.

(A) Expression of Tumor microenvironment related signatures were elevated in high (yellow) macrophage infiltration versus the low (blue) in IMvigor210. All statistics in the figure use two-sided Mann Whitney U test. $*p < 0.05$, $**p < 0.01$, $***p < 0.001$, $****p < 0.0001$. ns, not significant compared to isotype group.

(B) Metabolic signatures expression were significantly higher in low (blue) macrophage infiltration than those in high (yellow) in IMvigor210. All statistics in the figure use two-sided Mann Whitney U test. $*p < 0.05$, $**p < 0.01$, $***p < 0.001$, $****p < 0.0001$. ns, not significant compared to isotype group.

Figure S6

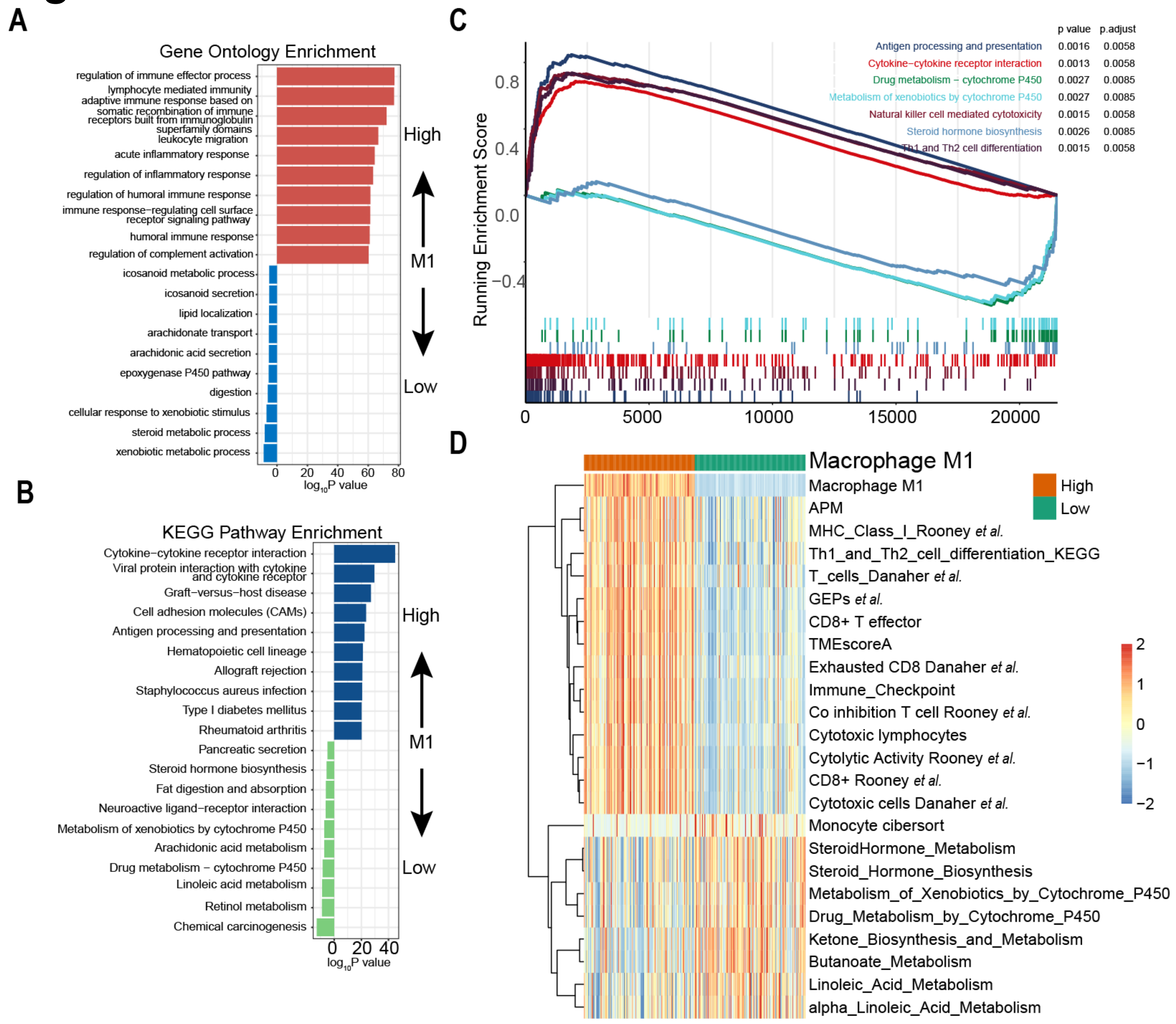

Transcriptomic and metabolic programs are reproducible in TCGA.

(A-B) Gene ontology (GO) (**A**) and KEGG pathways (**B**) were significantly associated with M1 macrophage infiltration in bladder TCGA data. Tumor with M1-deficient subtype have dramatically higher activation in steroid metabolism, xenobiotics metabolism whereas tumor with high-M1 infiltration embraced elevated immune activation. The top ten genes per set are shown (ranked by single-gene P value, GO: red: high, blue: low; KEGG: blue: high, green: low). Complete lists are given in Table S8.

(C) GSEA analyses of TCGA validated the key pathways enriched in high (up) and low (down) M1 subset. (light blue: metabolism of xenobolics by cytochrome P450; green: drug metabolism by cytochrome P450; blue violet: steroid hormone biosynthesis; scarlet: cytokine-cytokine receptor interaction; brick red: natural killer cell meditated cytotoxicity; dark violet: Th1 and Th2 cell differentiation; navy: antigen processing and presentation). Complete information and similar results of hallmark analyses are demonstrated in Table S8.

(D) Heatmap of unsupervised clustering different expressing gene signatures is consistent in (red: high expression; blue: low expression) in TCGA. Binary M1-macrophage infiltration was show as annotation on the top (red: high; green: low). Comprehensive information is displayed in Table S9.

Figure S7

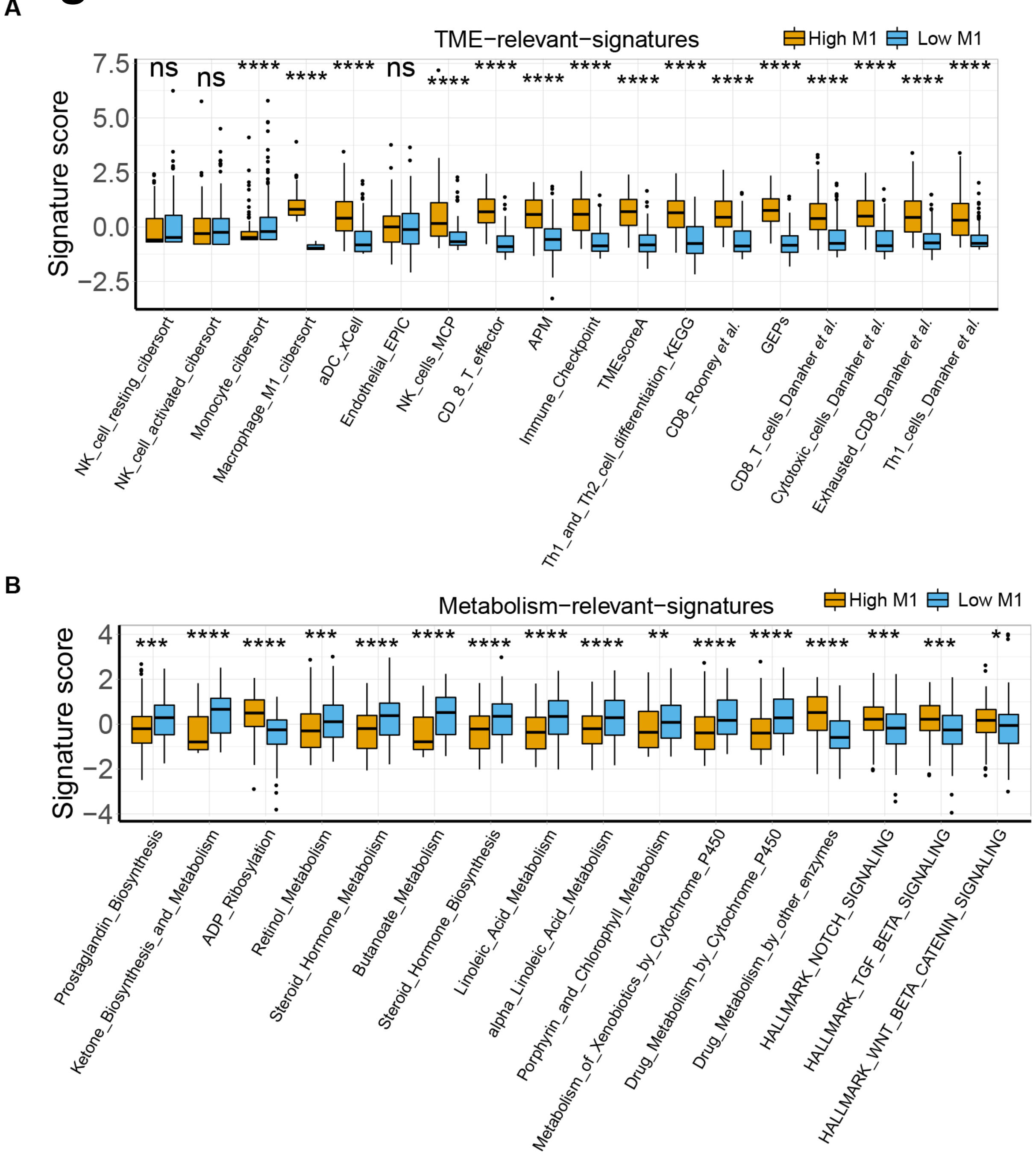

Validation of Tumor microenvironment and metabolic signatures profiles in TCGA.

(A) TCGA dataset externally validated that expression of Tumor microenvironment related signatures were elevated in high (yellow) macrophage infiltration versus the low (blue). All statistics in the figure use two-sided Mann Whitney U test. $*p < 0.05$, $**p < 0.01$, $***p < 0.001$, $****p < 0.0001$. ns, not significant compared to isotype group.

(B) External TCGA verified that Metabolic signatures expression were significantly higher in low (blue) macrophage infiltration than those in high (yellow). All statistics in the figure use two-sided Mann Whitney U test. $*p < 0.05$, $**p < 0.01$, $***p < 0.001$, $****p < 0.0001$. ns, not significant compared to isotype group.

Figure S8

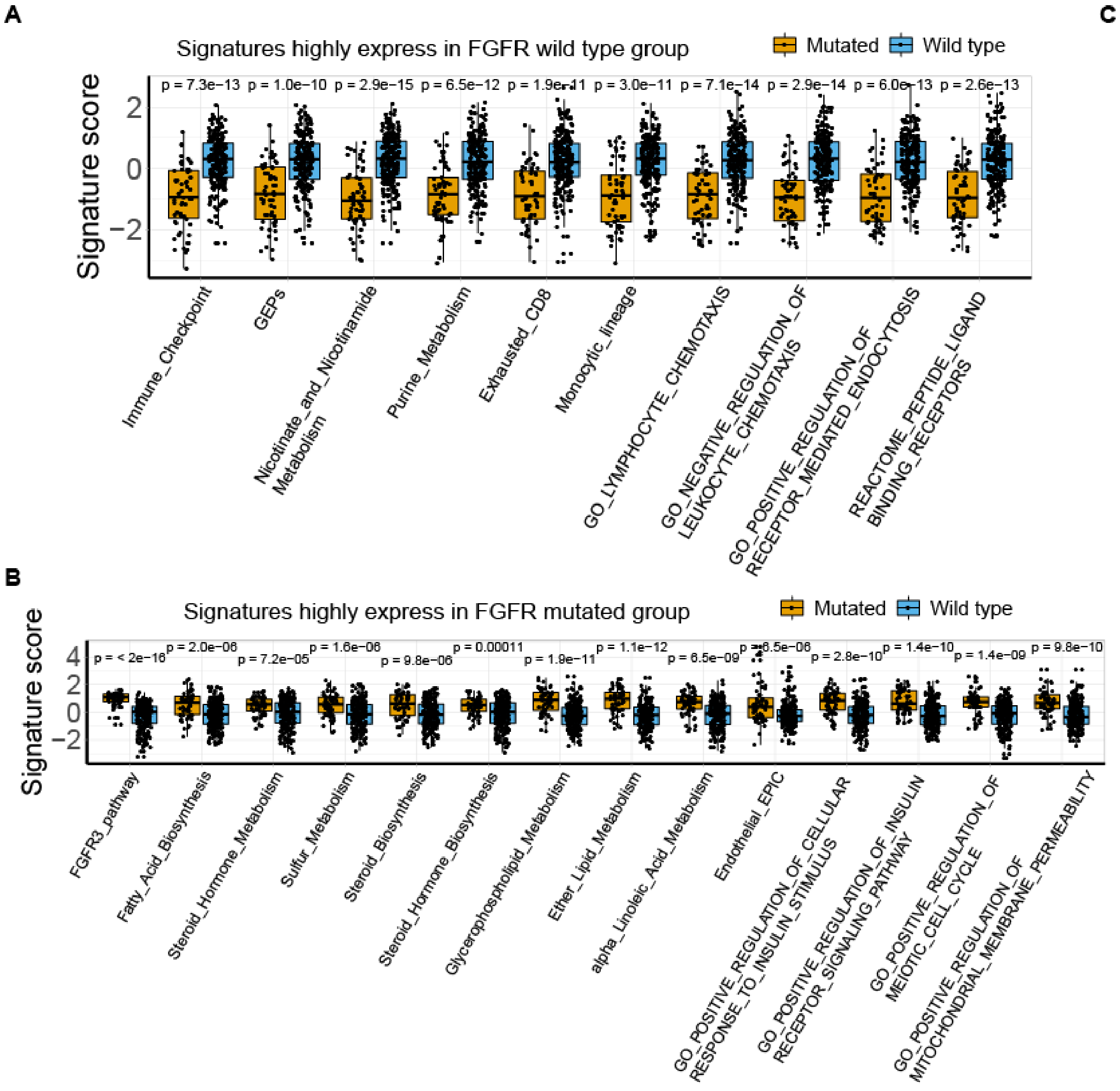

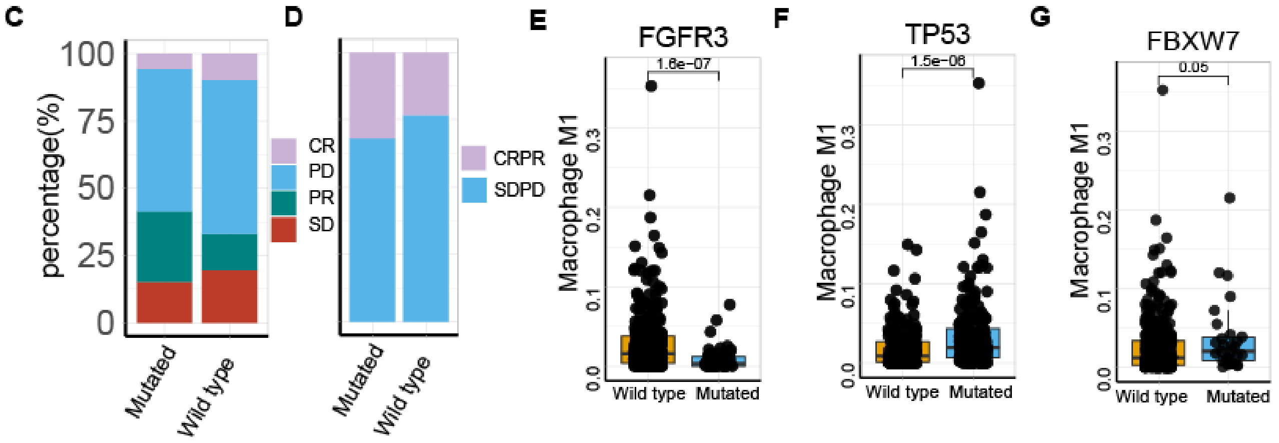

***FGFR* related signatures landscape and verification of genome in TCGA.**

(A-B) Signatures upregulated in *FGFR* mutation deficient subset (**A**) and mutated group (**B**) in IMvigor210 respectively. Steroid metabolism signatures are inclined to upregulated in *FGFR* mutated setting while T cell inflamed signatures and immune check point signatures in mutation-deficient fraction. Corresponding p values are shown.

(C-D) *FGFR* mutated group was linked with relative better response to anti-PD-L1 therapy. CR: complete response; PR: partial response; SD: stable disease; PD: progressive disease.

(E-G) External validation of M1 macrophage related DEGs mutation versus wild type in TCGA. *FGFR3* (**E**) *TP53* (**F**), *FBXW7* (**G**), (Mann Whitney U test, p = 1.6e-07, p = 1.5e-06; p = 5e-02, respectively).
